# Spatial heterogeneity in subglacial drainage driven by till erosion

**DOI:** 10.1098/rspa.2019.0259

**Published:** 2019-08-14

**Authors:** Indraneel Kasmalkar, Elisa Mantelli, Jenny Suckale

**Affiliations:** 1 Institute of Computational and Mathematical Engineering, Stanford University, Stanford, CA, USA; 2 Department of Geophysics, Stanford University, Stanford, CA, USA; 3 Department of Civil and Environmental Engineering, Stanford University, Stanford, CA, USA

**Keywords:** subglacial hydrology, subglacial processes, canals, ice streams, subglacial till, erosionalinstability

## Abstract

The distribution and drainage of meltwater at the base of glaciers sensitively affects fast ice flow. Previous studies suggest that thin meltwater films between the overlying ice and a hard-rock bed channelize into efficient drainage elements by melting the overlying ice. However, these studies do not account for the presence of soft deformable sediment observed underneath many West Antarctic ice streams, and the inextricable coupling that sediment exhibits with meltwater drainage. Our work presents an alternate mechanism for initiating drainage elements such as canals where meltwater films grow by eroding the sediment beneath. We conduct a linearized stability analysis on a meltwater film flowing over an erodible bed. We solve the Orr–Sommerfeld equation for the film flow, and we compute bed evolution with the Exner equation. We identify a regime where the coupled dynamics of hydrology and sediment transport drives a morphological instability that generates spatial heterogeneity at the bed. We show that this film instability operates at much faster time scales than the classical thermal instability proposed by Walder. We discuss the physics of the instability using the framework of ripple formation on erodible beds.

## 1. Introduction

Liquid water is present underneath more than half of the Antarctic Ice Sheet [1]. The hydrological environments in which this water is stored and transported are diverse, ranging from subglacial lakes to water-saturated wetlands situated underneath fast flowing ice [2]. Out of these, the drainage systems underneath ice streams, corridors of rapid ice flow that are tens of kilometres wide and hundreds of kilometres long, are not only the most spatially extensive but also inextricably coupled with the dynamics of the overlying ice flow [3–6]. While our understanding of subglacial drainage systems is incomplete, it is becoming increasingly clear that these drainage systems are both spatially and temporally variable [4,7].

Water flow, however, is not the only dynamic component in the extensive wetlands underneath the West Antarctic ice streams. Large portions of this area rest on weak and unconsolidated sediment, commonly referred to as till [8]. Samples collected from the subglacial environment at Ice Stream B, Siple Coast, have revealed a fine-grained, clay-rich lithology [9] that likely experiences significant deformation [10–12] and transport [10] due to streaming ice flow.

The insight that sediments play an important role in subglacial hydrology and ice flow is not new, and several previous models of subglacial hydrology have made progress in that regard. Early attempts treated the subglacial horizon as an aquifer with porous flow being the primary means of water drainage [13,14]. While percolation of water into the till is important in altering the basal resistance that the till layer provides to ice flow [15,16], water transport through the till is likely very inefficient [17] because of the low permeability of clay-rich till [11,18]. Later models have replaced the idea of Darcian-type water transport through a porous aquifer by assuming that most of the water flows in a thin pressurized film between the ice and the till [17,19–21].

Walder [22] identified a problem with large-scale water transport via film flow by pointing out that meltwater films over hard beds are fundamentally unstable. His work [22] highlights that a small perturbation in film thickness would lead to higher water flux, which would induce greater viscous dissipation and preferential melting of the overlying ice until the melting is balanced by creep closure of the ice. Creyts & Schoof [23] later showed that this instability is partially suppressed by bed roughness, reinvigorating the idea that films could support meltwater transport at least up to a certain thickness. They argue that stress localization on bed protrusions leads to enhanced ice roof closure that counters film expansion, thus entailing the possibility of finite-sized films. Nonetheless, as the thickness of the film grows, the water would eventually carve into the ice via melting, thus transforming the film into a more efficient drainage element such as a Röthlisberger channel [24] or a linked cavity [25]. This insight is reflected in current subglacial hydrology models for hard beds that generally include both films and channels [26–30].

In hard-bed settings, efficient drainage systems will inevitably be carved into the ice [25]. The widespread occurrence of till under ice sheets [9,31] suggests the possibility of drainage elements incised into the sediment layer, such as canals [32–36]. Walder & Fowler [32] show that dynamic till, in particular the processes of till erosion and deformation, is key to the sustenance of canals. Ng [33] builds on the work by Walder and Fowler by describing the meltwater flux and sediment transport dynamics over the longitudinal span of a canal. Given the importance of the coupled processes of meltwater and till in the sustenance of canals, it is likely that these processes also play key roles in the formation of such canals. Departing from the classical idea of Walder's instability [22] that films grow into channels by melting the ice above, we hypothesize that meltwater films on soft beds generate a spatially heterogeneous drainage system by eroding the sediment beneath.

Kyrke-Smith & Fowler [21] have previously studied the evolution of meltwater films on soft beds. They emphasize the role of dynamic till by explicitly including erosion and deformation into their model. However, they retain the assumption of static bed roughness from the hard-bed setting to stabilize thin films [23]. Our work provides an alternative framework for the stability of meltwater films that describes the formation of bed roughness as a dynamic process resulting from the coupling between film hydrology and sediment transport.

We model the film as flow over an erodible bed and study morphological instabilities of the system, similar to granular ripple formation (e.g. [37–40]). We compute bed-form evolution using the classical Exner equation that represents sediment mass conservation. We use the three-dimensional Navier–Stokes equations to compute flow velocities within the film. A depth-averaged velocity approach, while commonly used for meltwater films [25], is not suitable for morphological instabilities because of the lack of resolution of near-bed flow dynamics [41,42].

Walder's instability of film expansion via dissipation-induced melting of the ice is known to drive channel initiation [19,22]. To identify potential instabilities that may occur prior to Walder's instability, we assume non-turbulent flow within the meltwater film. This assumption allows us to study the film within a regime where dissipation and associated ice melt is mitigated. In this regime, and over length scales comparable to film thickness, the overlying ice is effectively decoupled from the film hydrology. Our set-up then allows us to explore instabilities associated with the sediment bed rather than the ice.

We conduct a linearized stability analysis of the system. We find that water transport in a thin film destabilizes the bed. The underlying physics of this bed instability is similar to that of ripple formation [37,43]. Unlike ripples, however, we show that the structure generated by the instability has a component transverse to the main flow. As a result, a spatially heterogeneous drainage element emerges from the bed. While we do not study its temporal evolution explicitly, the spatially heterogeneous drainage could potentially evolve into a well-defined drainage element such as a canal or a system of linked cavities.

Our study also identifies a hydrodynamic mechanism that suppresses short-wavelength structures at the bed. By contrast, prior studies attribute the stability of short-wavelength structures to diffusive mechanisms within the sediment transport dynamics of the film [40]. While sediment-based mechanisms may provide additional stability to the system, our work shows that they are not necessary for the selection of the fastest growing perturbation wavelength.

## 2. Model

**Figure 1.**
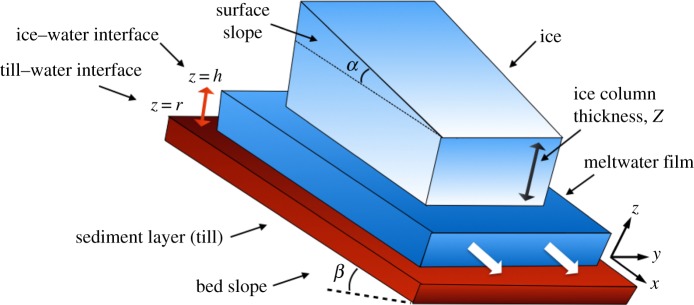
Set-up of the model. The thicknesses of the three layers are not drawn to scale. The ice column is several orders of magnitude thicker than the meltwater film. (Online version in colour.)

We consider a thin layer of subglacial meltwater, flowing between two initially homogeneous, infinitely extended layers of ice and till on the top and bottom, respectively, both inclined at an angle *β* with respect to the horizontal. The surface of the ice possesses its own slope, *α* with respect to its base. We adopt a Cartesian coordinate system (*x*, *y*, *z*). As shown in figure 1, the *x*-axis is parallel to the bed and denotes the along-flow direction, while the *y*- and *z*-axes span the cross-flow direction and the depth of the film. We represent the ice–water interface by *z* = *h*(*x*, *y*, *t*) and the till–water interface by *z* = *r*(*x*, *y*, *t*).

Our model includes two components: fluid flow, described by mass and momentum conservation; and sediment transport, which governs the evolution of the till–water interface. We discuss the thermal and mechanical interactions of ice and water, and revisit the underlying assumptions in §4. The key thermal interaction between ice and water lies in the energy budget at the corresponding interface, namely the melting of the ice caused by the heat flux from the film. We assume a non-turbulent flow regime where the dissipation-induced melting of the ice is suppressed. Combined with the assumption that sediment transport processes are significantly faster than ice-related processes, we treat the ice–water interface as a fixed boundary. The key mechanical interaction between ice and water is the pressure exerted on the film by the weight of the ice column. The corresponding pressure gradient serves as a driving force for the film flow.

We now present the governing equations of the meltwater film system.

### (a) Hydrology

Conservation of mass within the subglacial meltwater film, along with incompressibility, yields

2.1 ∇⋅u=0,on r<z<h,

where ***u*** = (*u*, *v*, *w*) is the fluid velocity along the axes (*x*, *y*, *z*), respectively.

The Navier–Stokes equations express momentum conservation within the meltwater film

2.2 $$\frac{\partial u}{\partial t}+u\cdot \nabla u=-\frac{1}{\rho }\nabla p+\nu \nabla^{2}u+g,\;\text{on}\;r<z<h,$$

where *t* stands for time, *ν* is the kinematic viscosity of water at the melting point and *ρ* is the density of water, *p* is the fluid pressure, ***g*** = (*g*sin*β*, 0 − *g*cos*β*) is the gravitational force, and *g* is the gravitational acceleration on the Earth's surface.

We assume that the ice–water interface is a fixed boundary and apply the no-slip condition

2.3 u=0,v=0,w=0,at z=h.

The balance of normal stresses at the ice–water interface is given by

2.4 $$p=p_{a}+(Z_{0}+Z_{1}(x))\rho_{i}g\cos\beta ,\;\text{at}\;z=h,$$

where *p*_*a*_ is the atmospheric pressure at the surface of the ice and *ρ*_*i*_ is the density of ice. The term *Z*_0_ is the leading-order ice thickness measured perpendicular to the bed. At length scales comparable to film thickness, *Z*_0_ is constant, indicating that the ice surface is parallel to the bed. The term *Z*_1_(*x*) represents change in the ice thickness that occurs at scales comparable to film thickness. In (2.4), the fluid normal stress at the ice–water interface reduces to the pressure *p* as a consequence of (2.1) and (2.3). We approximate the normal stress imposed by the ice on the film by the weight of the overlying ice column. This approximation follows from the Shallow Ice assumption, namely that the ice thickness is considerably smaller than the ice sheet length scale.

We assume a no-slip boundary condition at the till–water interface. Since the bed evolves over time, the vertical velocity satisfies the kinematic boundary condition

2.5 $$u=0,\;v=0,\;w=\frac{\partial r}{\partial t},\;\text{at}\;z=r.$$

The no-slip boundary condition (2.5) is an approximation of a permeable boundary that separates the film and the wet sediment. This approximation is likely not strictly true in nature, but reasonable if the fluid speed in the till is significantly slower than in the film. The no-slip condition is commonly used for stability analyses of films over erodible beds (e.g. [41,43,44]).

### (b) Sediment bed-load transport

We model the evolution of the till–water interface *z* = *r*(*x*, *y*, *t*) through the Exner equation

2.6 $$\frac{\partial r}{\partial t}+\frac{1}{1-\phi_{m}}\nabla \cdot q=0,$$

where *ϕ*_*m*_ is the mean sediment porosity and ***q*** = (*q*_1_, *q*_2_) is the sediment flux vector. The Exner equation is a mass conservation statement for the sediment layer, stating that the till–water interface evolves in time according to the divergence of the sediment flux.

To close the model, we use a classical constitutive relation that expresses the sediment flux ***q*** as a function of the shear stress applied by the water film onto the bed [45–47]

2.7 $$q=\hat{\tau }F\left(\frac{|\tau |}{(\rho_{s}-\rho )gD}\right)D\sqrt{\frac{\rho_{s}-\rho }{\rho }gD},$$

where *D* is the sediment grain diameter, *ρ*_*s*_ is the sediment density and *F* is a non-dimensional function to be defined later. The bed stress vector, **τ** = (τ_1_, τ_2_), and its unit vector, $\hat{\tau }$, are given by

2.8 $$\tau_{i}=\rho \nu t_{i}^{T}\left(\nabla u+\nabla u^{T}\right)n,\;\hat{\tau }=\frac{\tau }{|\tau |},\;\text{at}\;z=r.$$

The vectors ***t***_1_ and ***t***_2_ are the unit tangent vectors to the bed in the *x*- and *y*-directions, respectively, and ***n*** is the unit normal to the bed *z* = *r* (see the electronic supplementary material for more details).

There is considerable disagreement about the functional form relating the sediment flux to the bed shear stress (e.g. [48–50]). As a result, there are a variety of empirically derived power-law formulae in the literature, each calibrated to its own setting (e.g. [46,47]). This diversity of forms highlights that the physics of bed-load transport is not yet fully understood.

Observations of bed-load transport highlight that there is a threshold value of bed stress below which there is purportedly no grain motion at the bed, as discussed in the classical work by Shields [48] and others (e.g. [51–53]). Houssais *et al.* [53] suggest that this threshold value, known as the critical Shields stress, marks a phase transition for the granular material. Below the threshold the bed stress is balanced by extremely slow creep deep in the sediment, while exceeding the threshold leads to the formation of an overlying bed-load layer undergoing dense granular flow at a time scale comparable to that of near-bed fluid flow. Experimental studies [52–54] characterize this phase transition as a toggle point, where bed-load transport is deemed active only when the fluid bed stress exceeds the threshold stress. Our model uses the threshold in the same manner and we assume that the film bed stress always exceeds this threshold. This assumption is a key prerequisite for the linearized stability analysis conducted in §5.

For the purposes of this study, we choose the classic Meyer-Peter & Müller [45] functional

2.9 $$F\left(\frac{|\tau |}{\left[\tau_{b}\right]}\right)=\{\begin{array}{cc} A{\left(\frac{|\tau |}{\left[\tau_{b}\right]}-\tau_{c}^{*}\right)}^{m}\; & \frac{|\tau |}{\left[\tau_{b}\right]}\geq \tau_{c}^{*}\;\\ 0\; & \frac{|\tau |}{\left[\tau_{b}\right]}<\tau_{c}^{*}\; \end{array},\;A=8,\;m=1.5,$$

where [**τ**_*b*_] = (*ρ*_*s*_ − *ρ*)*gD* is the associated scale for bed stress, and τ_c_^*^ is the non-dimensional critical Shields stress. Experiments for non-turbulent flow over erodible beds suggest that τ_c_^*^ = 0.12 [52,54]. We do not include sediment suspension in our model since the lack of turbulence precludes the possibility of sediment saltation (e.g. [52,55,56]).

We choose a specific functional for the purposes of our analysis, but our approach can be repeated for a broad range of functionals satisfying (2.7). The linearized stability analysis conducted in §5 demonstrates that the exact choice of functional does not alter the overall stability of the meltwater film. Insensitivity to the functional broadens the applicability of our model to a wide variety of glaciological settings.

### (c) Steady state

We solve the system of equations (2.1)–(2.6) for the steady state. We assume that the steady-state solution is uniform in the *x*- and *y*-directions.

The ice topography imposes a driving force on the fluid, as described in (2.4). The pressure gradient within the film arises from the change in ice thickness, (∂*Z*_1_/∂*x*) that occurs over a length comparable to meltwater film thickness. We parametrize this term as

2.10 $$\frac{\partial Z_{1}}{\partial x}=-\tan\alpha ,$$

where *α* represents the slope of the ice surface with respect to the bed. We combine both the driving forces, gravity (2.2) and ice overburden (2.4), into a single parameter

2.11 $$\pi =\sigma_{i}\tan\alpha \cos\beta +\sin\beta ,\;\text{where}\;\sigma_{i}=\frac{\rho_{i}}{\rho }.$$

The steady-state solution below describes a Poiseuille flow profile with uniform bed-load transport. We denote steady-state variables by an overlying bar. We define *H* as half the film thickness at steady state. The constant of half allows us to avoid re-scaling in other equations

2.12 $$\begin{array}{rl} \bar{h}(x,y) & =2H,\;\bar{r}(x,y)=0, \end{array}$$

2.13 $$\begin{array}{rl} \bar{u}(x,y,z) & =\frac{H^{2}g\pi }{2\nu }\frac{z}{H}\left(2-\frac{z}{H}\right),\;\bar{v}=0,\;\bar{w}=0,\;\text{on}\;\bar{r}\leq z\leq \bar{h}, \end{array}$$

2.14 $$\begin{array}{rl} \bar{p}(x,y,z) & =p_{a}+\rho_{i}gZ_{0}\cos\beta -\rho gx\tan\alpha \cos\beta ,\;\text{on}\;\bar{r}\leq z\leq \bar{h}, \end{array}$$

2.15 $$\begin{array}{rl} {\bar{\tau }}_{1}(x,y) & =\rho gH\pi ,\;{\bar{\tau }}_{2}(x,y)=0 \end{array}$$

2.16 $$\begin{array}{rl} \mathrm{and}\;\;{\bar{q}}_{1}(x,y) & =F\left(\frac{|\bar{\tau }|}{(\rho_{s}-\rho )gD}\right)D\sqrt{\frac{\rho_{s}-\rho }{\rho }gD},\;{\bar{q}}_{2}(x,y)=0. \end{array}$$

## 3. Non-dimensionalization and simplification

We list the main variables and define their scales (denoted by square brackets) in table 1.

**Table 1.** Characteristic scales of system variables.

variable	scale	description
[*x*], [*y*], [*z*]	*H*	The coordinate system scales with the film thickness.
[*h*]	*H*	The ice–water interface, which is considered a fixed boundary, is at distance 2*H* from the till–water boundary.
[*r*]	*D*	The evolution of the till–water interface is governed by an active bed-load layer that is a few grain diameters in thickness [53,56].
[***u***]	$\frac{H^{2}g\pi }{2\nu }$	The velocity scale is derived from (2.13).
[***τ***]	*ρgH*π	The bed stress scale is derived from (2.8).
[*p*]	*ρgH*	This scale is consistent with the hydrostatic pressure in (2.14).
[***q***]	$\frac{\rho_{s}}{\rho }D\frac{D}{H}[u]$	Experiments by Houssais *et al*. [53] suggest that ***q*** scales with grain density (∼*σ*), bed-load layer thickness (∼*D*) and near-bed fluid velocity $\left(∼\frac{D}{H}[u]\right)$.
[*t*]	$(1-\phi_{m})\frac{DH}{\left[q\right]}$	This characteristic time scale is defined according to the Exner equation (2.6) and describes the rate of sediment transport.

Denoting the non-dimensionalized variables with stars, the non-dimensional forms of equations (2.1)–(2.9) are given by

3.1 $$\begin{array}{rl} \nabla \cdot u^{*} & =0,\;\text{on}\;r^{*}<z^{*}<2, \end{array}$$

3.2 $$\begin{array}{rl} \gamma \frac{\partial u^{*}}{\partial t^{*}} & =-u^{*}\cdot \nabla u^{*}+\frac{1}{Re}\left[\nabla^{2}u^{*}-\frac{2}{\pi }\nabla p^{*}+\frac{2}{\pi }g^{*}\right],\;\text{on}\;r^{*}<z^{*}<2, \end{array}$$

3.3 $$\begin{array}{rl} \frac{\partial r^{*}}{\partial t^{*}} & =-\nabla \cdot q^{*},\;q^{*}=\kappa F(S|\tau^{*}|)\hat{\tau },\;F(S|\tau^{*}|)=A{\left(S|\tau^{*}|-{\tau_{c}}^{*}\right)}^{m}, \end{array}$$

3.4 $$\begin{array}{rl} u^{*} & =0,\;p^{*}=\frac{p_{a}}{\rho gH}+\sigma_{i}\frac{Z_{0}}{H}\cos\beta -\sigma_{i}x^{*}\cos\beta \tan\alpha ,\;\text{at}\;z^{*}=2 \end{array}$$

3.5 $$\begin{array}{rl} \mathrm{and}\;\;u^{*} & =0,\;v^{*}=0,\;w^{*}=L\gamma \frac{\partial r^{*}}{\partial t^{*}},\;\text{at}\;z^{*}=Lr^{*}, \end{array}$$

where g* = (sin*β*, 0,  − cos*β*), *Re* is the Reynolds number, *L* is the grain-to-film size ratio, *σ* is the grain-to-fluid density ratio, *γ* is the hydrology-to-sediment transport time-scale ratio, *S* is the steady-state non-dimensional bed stress, also known as Shields number, and *κ* is a non-dimensional constant for the bed-load functional

3.6 $$L=\frac{D}{H},\;Re=\frac{[u]H}{\nu },\;\sigma =\frac{\rho_{s}}{\rho },\;\gamma =\frac{H}{\left[u][t\right]},\;S=\frac{\left[\tau \right]}{(\rho_{s}-\rho )gD},\;\kappa =\frac{D\sqrt{(\sigma -1)gD}}{\left[q\right]}.$$

The main dimensional parameters of the system, *H*, *D*, *β*, *α*, π, *ρ*, *ρ*_*s*_, *ρ*_*i*_, *ϕ*_*m*_, *g*, *ν* and τ_c_^*^, reduce to the following independent dimensionless quantities: *Re*, *L*, *β*, *α*, *σ*, *σ*_*i*_, *ϕ*_*m*_ and τ_c_^*^. We do not include the terms *p*_*a*_ and *Z*_0_ as parameters since they only contribute to the ambient pressure in (3.4) and do not affect the dynamics of the system. The dependent dimensionless quantities are given by

3.7 $$\gamma =\frac{L\sigma }{1-\phi_{m}},\;S=\frac{\prod }{(\sigma -1)L},\;\kappa =\frac{\sqrt{2(\sigma -1)}}{\sigma \sqrt{ReL\prod }},\;\prod =\sigma_{i}\tan\alpha \cos\beta +\sin\beta .$$

Among the eight independent non-dimensional quantities, the latter four, *σ*, *σ*_*i*_, *ϕ*_*m*_ and τ_c_^*^, tend to vary by less than an order of magnitude over the range of subglacial settings. We represent them with constant values as given in table 2. We also assume that the bed slope and surface slope are roughly comparable, i.e. *β*∼*α* [57,58]. This allows us to simplify π in (2.11)

3.8 ∏≈2sinα.

In summary, the system is determined by three non-dimensional parameters *Re*, *L* and *α*.

**Table 2.** Fundamental parameters of the model, along with their estimates and ranges.

parameter	estimate/range	description
*H*	10^−4^ m ≤ *H* ≤ 10^−2^ m	Film thickness (divided by 2). Values based on observations [59] and drainage theory [23].
*D*	10^−6^ m ≤ *D* ≤ 10^−4^ m	Grain diameter. Core measurements [31] reveal a bi-modal clay (*D*∼1 μm) and sand distribution (*D*∼100 μm).
*β*	10^−4^ ≤ *β* ≤ 0.1	Bed slope angle. Values from [57]. The large range allows our model to consider both the polar and alpine settings.
*g*	9.8 ms^−2^	Gravitational acceleration near the Earth's surface.
*ν*	1.787 × 10^−6^ m^2^s	Kinematic viscosity of water at 0°C.
*ρ*	1000 kgm^−3^	Density of water at 0°C.
*ρ*_*s*_	2600 kgm^−3^	Density of sediment grains, assuming clay-like material.
*ρ*_*i*_	917 kgm^−3^	Density of ice.
*ϕ*_*m*_	0.4	Mean porosity of subglacial sediment [9].
τ_c_^*^	0.12	Threshold Shields stress. Empirical value [52–54].
*σ*	2.6	Grain-to-fluid density ratio.
*L*	10^−4^ ≤ *L* ≤ 10^−2^	Grain-to-film size ratio. Our model assumes *L*≪1.
*Re*	*Re* < 10^4^	Reynolds number. We assume a non-turbulent regime.
*α*	10^−4^ ≤ *α* ≤ 0.1	Ice surface slope angle. Values from [57].

We assume that sediment grains are very small compared with the film size, i.e. *L*≪1. Equation (3.7) then implies *γ*≪1 and reduces (3.5) to homogenous boundary conditions

3.9 $$w^{*}=L\gamma \frac{\partial r^{*}}{\partial t^{*}}=O(L^{2})∼0,\;\text{at}\;z=r.$$

The electronic supplementary material provides the mathematical details underlying (3.9).

The assumption that the system is always above the threshold stress value in (2.7), is given by

3.10 S>0.12.

## 4. Applicability of the model

Since glacial settings are diverse, it is valuable to clarify where the assumptions and scaling choices within our model are applicable. In this section, we identify regions of the parameter space that lie within the scope of our model.

**Figure 2.**
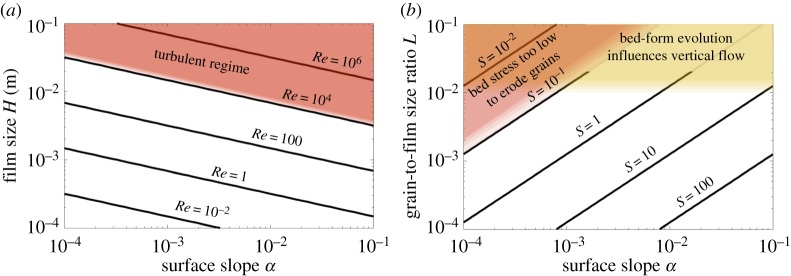
(*a*) Contour plot of Reynolds Number *Re* for a given film size *H* and surface slope *α*. (*b*) Contour plot of the non-dimensional bed stress Shields Number *S* for a given grain-to-film size ratio *L* and surface slope *α*. The model is inapplicable within the shaded regions. (Online version in colour.)

One of the key requirements of our model is maintaining a low-to-intermediate Reynolds number for the film flow (*Re* < 10^4^). Since the Reynolds number, defined in (3.6), is governed by the film thickness scale *H* and the surface slope *α*, due to (3.8), we plot contours of *Re* against these parameters in figure 2*a*. The range of surface slopes aims to capture both the polar setting, especially the Siple Coast, West Antarctica (e.g. *α*∼10^−3^ [57]) as well as the alpine setting which is characterized by steeper slopes. The red-shaded region highlights *Re* > 10^4^, which we consider as the turbulent regime. Our model is only applicable to non-turbulent films, which are of the order of centimetres in thickness or less, as per figure 2*a*. Observational evidence records films with thicknesses of 1 μm to 0.1 *mm* [60,61], which is on the lower end of our parameter space. Theoretical studies generally assume films that are millimetres thick [22,23].

Active sediment transport at the bed of the film is a key prerequisite for potential channelization. This requirement is described by the bed stress exceeding the critical Shields stress needed to erode sediment grains (3.10). Within our model, the non-dimensional bed stress *S* is a function of surface slope *α* and grain-to-film size ratio *L*, described in (3.6). Figure 2*b* shows the contour plots of *S* over the ranges of *α* and *L*. The region where the bed stress does not exceed the critical Shields threshold is represented by the red-shaded triangle.

The parameter *L* also governs feedbacks between the bed and the vertical flow velocity as represented in the kinematic boundary condition (2.5). The assumption *L*≪1 enables us to ignore kinematic boundary effects in (3.9). The yellow-shaded rectangle in figure 2*b* highlights the region of *L* > 10^−2^, which is beyond the scope of our model.

### (a) Competition between ice melt and sediment transport

Since our model focuses on the mechanical coupling of water and sediment while excluding thermal processes associated with the ice, its applicability is restricted to a regime where sediment transport is significantly faster than film-induced melting of the ice. We conduct a rudimentary comparison of time scales to identify the parameter space that characterizes this regime. We introduce a simple thermal model for melting of the ice. Note that the presence of a subglacial meltwater film indicates a temperate basal setting, i.e. the base of the ice is at melting point. The energy balance at the ice–water interface is described by the Stefan equation

4.1 $$L_{H}\rho_{i}\frac{\partial h}{\partial t}=Q^{+}-Q^{-},$$

where *L*_*H*_ = 3.36 · 10^5^ J kg^−1^ is the latent heat of fusion of water, *Q*^+^ is the heat flux into the ice from the water along the direction normal to the interface, and *Q*^−^ is the heat outflux.

The potential sources of heat influx *Q*^+^ for subglacial settings are frictional heating of ice sliding over the bed, film thermal dissipation and geothermal heat flux [58]. Frictional heating is suppressed in the presence of a meltwater film which lubricates the ice-bed contact. Thermal dissipation within the film is negligible in non-turbulent settings. The main source of heat flux in our setting is geothermal, which is transported through the film to the ice–water interface. We assume that the heat influx *Q*^+^ scales with the geothermal heat flux *G*, i.e. *Q*^+^∼*G*.

The heat outflux at the ice–water interface is a result of conduction through the ice. With the goal of deriving a conservative upper bound on the ice melt, we assume that the outflux is negligible compared with the influx, i.e. *Q*^−^ = 0. We derive the ice melt time scale [*t*_*i*_] using (4.1)

4.2 $$L_{H}\rho_{i}\frac{D}{\left[t_{i}\right]}=G.$$

We scale the evolution of the ice–water interface *h* by the sediment grain size *D* to make an appropriate comparison with the erosion-based evolution of the till–water interface.

The Exner equation (2.6) and Stefan equation (4.1) highlight two processes for the evolution of a meltwater film. We compare the time scales of these two processes, namely ice melt and sediment transport, to identify which process is faster at carving out a channel. We represent the ratio of the time scales of these two processes, *R* = ([*t*]/[*t*_*i*_]), defined in (4.2) and table 1

4.3 $$R=\frac{\nu (1-\phi_{m})G}{\sigma D^{2}L_{H}\rho_{i}g\sin\alpha }.$$

**Figure 3.**
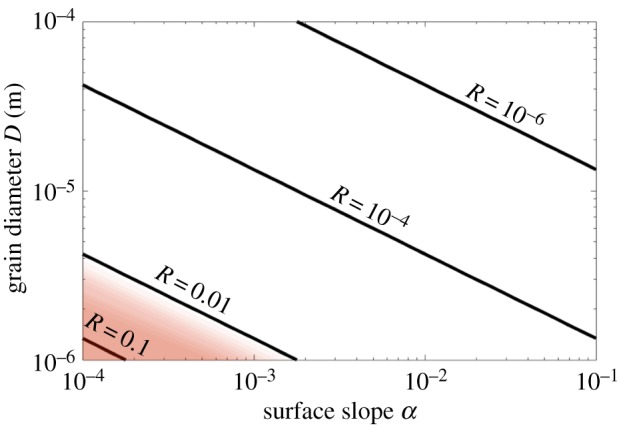
Contour plots of the ratio of time scales, defined in (4.3). Geothermal heat flux *G* = 0.13 Wm^−2^. The red-shaded region indicates *R* > 0.01 where the two time scales are comparable. (Online version in colour.)

We calculate *R* over our parameter space. Observational estimates for geothermal heat flux in the Siple coast region show a range of 0.04 Wm^−2^ ≤ *G* ≤ 0.13 Wm^−2^ [62]. Since the variation in *G* is less than an order of magnitude, we choose a representative value, *G* = 0.13 Wm^−2^. This higher end value provides a conservative upper bound for the rate of ice melt. We plot *R* as a function of surface slope *α* and grain diameter *D* in figure 3. The figure indicates sediment transport is several orders of magnitude faster than ice melt for most of the parameter space. The red-shaded triangle denotes the region where *R* is close to 1, indicating that the model is inapplicable within the region. Overall, figure 3 suggests that decoupling the dynamics of the ice–water interface from the meltwater film for the purposes of stability analysis is a suitable assumption for a large variety of glaciological settings.

## 5. Linearized stability analysis

To better understand the stability of a subglacial meltwater film flowing over a sediment bed, we perform a linearized stability analysis on the model presented in §3.

We consider small amplitude perturbations about the steady state (2.13) and expand dependent non-dimensional variables as

5.1 $$f(x,y,z,t)=\bar{f}(z)+\varepsilon \tilde{f}(x,y,z,t),\;\varepsilon \ll 1,$$

where barred quantities represent steady-state variables, and for simplicity we have omitted stars denoting non-dimensional variables. In the light of the domain being infinitely extended in the *x*- and *y*-directions, we represent the perturbation as elements of the Fourier basis

5.2 $$\tilde{f}(x,y,z,t)=\hat{f}(z,t)\exp\left(ik_{1}x+ik_{2}y\right),$$

where *k*_1_ and *k*_2_ are the wavenumbers of the perturbations in the *x*- and *y*-directions, respectively. Given the linearity of the system, we also assume separation of variables in *z* and *t*

5.3 $$\hat{f}(z,t)=f^{\prime }(z)\exp\left(\omega t\right),\;\omega =\omega_{r}+i\omega_{i},$$

where *ω*_r_ and *ω*_i_ are the real and imaginary parts of *ω*, respectively. Note that ***q*** and *r* do not vary along the depth, hence ***q***′ and *r*′ are constants. The variable *r*′ represents the amplitude and phase of the bed-form perturbation. Given the linearity of the system, the variables *u*′, *v*′, *w*′ and *p*′ scale linearly with *r*′ while *ω* is independent of *r*′. When performing computations, we set *r*′ = 1.

The sign of *ω*_r_ is the key indicator of stability within the meltwater film. If *ω*_r_ > 0, then the perturbation in the system described by wavenumbers (*k*_1_, *k*_2_) undergoes exponential amplification with time, indicating unstable growth of the bed-form. The goal of the linearized stability analysis is to compute *ω*_r_ given model parameters and perturbation wavenumbers *k*_1_, *k*_2_. If there exists some pair (*k*_1_, *k*_2_) for which *ω*_r_ > 0, then the system is deemed unstable.

We introduce small-amplitude perturbations described in (5.1) and linearize the equations (3.1)–(3.5) around the steady state. Denoting the derivative $\frac{d}{dz}$ by *D*, we obtain the equations

5.4 $$\begin{array}{rl} 0 & =ik_{1}u^{\prime }+ik_{2}v^{\prime }+Dw^{\prime },\;\text{on}\;0<z<2, \end{array}$$

5.5 $$\begin{array}{rl} \gamma \omega u^{\prime } & =-ik_{1}\bar{u}u^{\prime }-wD\bar{u}-\frac{2ik_{1}}{\prod Re}p^{\prime }+\frac{1}{Re}\left[-k_{1}^{2}-k_{2}^{2}+D^{2}\right]u^{\prime },\;\text{on}\;0<z<2, \end{array}$$

5.6 $$\begin{array}{rl} \gamma \omega v^{\prime } & =-ik_{1}\bar{u}v^{\prime }-\frac{2ik_{2}}{\prod Re}p^{\prime }+\frac{1}{Re}\left[-k_{1}^{2}-k_{2}^{2}+D^{2}\right]v^{\prime },\;\text{on}\;0<z<2, \end{array}$$

5.7 $$\begin{array}{rl} \gamma \omega w^{\prime } & =-ik_{1}\bar{u}w^{\prime }-\frac{2}{\prod Re}Dp^{\prime }+\frac{1}{Re}\left[-k_{1}^{2}-k_{2}^{2}+D^{2}\right]w^{\prime },\;\text{on}\;0<z<2, \end{array}$$

5.8 $$\begin{array}{rl} 0 & =u^{\prime },\;0=v^{\prime }\;0=w^{\prime },\;0=p^{\prime },\;\text{at}\;z=2, \end{array}$$

5.9 $$\begin{array}{rl} 0 & =u^{\prime }+Lr^{\prime }D\bar{u},\;0=v^{\prime },\;0=w^{\prime },\;\text{at}\;z=0 \end{array}$$

5.10 $$\begin{array}{rl} \mathrm{and}\;\;\omega r^{\prime } & =-ik_{1}S\kappa d\bar{F}Du^{\prime }-ik_{2}\kappa \bar{F}Dv^{\prime },\;\text{at}\;z=0, \end{array}$$

where $\bar{F}$ and $d\bar{F}$ are steady-state sediment transport values derived via (3.3) for $\bar{\tau }=(1,0)$,

5.11 $$\bar{F}=F(S|\bar{\tau }|)=A{\left(S-0.12\right)}^{m},\;d\bar{F}=\frac{dF}{d|\tau |}(S|\bar{\tau }|)=Am{\left(S-0.12\right)}^{m-1},\;A=8,\;m=1.5.$$

We derive the linearization of the Exner equation (5.10) in the electronic supplementary material.

Since the bed-load transport functional (2.9) is non-differentiable at the threshold for initiating grain erosion, condition (3.10) is mathematically necessary to ensure that we can linearize the system of equations for the perturbations around the steady state.

In (5.9), the no-slip boundary conditions (3.5) at the moving boundary *z* = *r* have been transformed by a Taylor expansion in *ε* into equivalent boundary conditions imposed at the domain boundary *z* = 0. The equivalence allows us to solve the system of equations on a fixed domain while obtaining the solution to the original problem with an evolving till–water interface.

### (a) Reformulation in terms of streamfunction

The perturbation introduced into the steady state is two-dimensional in nature, described by the wavenumbers *k*_1_ and *k*_2_. To simplify our analysis, we reduce the perturbation to a single dimension. The Squire transformation [63–65] is a classical method that projects three-dimensional fluid flow onto a plane while preserving its perturbation characteristics. This plane, known as the Squire plane, is defined by the *z*-axis, and the vector (*k*_1_, *k*_2_) in the horizontal plane. We define *k* as the Squire perturbation wavenumber, *θ* as the Squire angle, and U as the horizontal velocity in the (*k*_1_, *k*_2_) direction, such that

5.12 $$k_{1}=k\sin\theta ,\;k_{2}=k\cos\theta ,\;kU^{\prime }=k_{1}u^{\prime }+k_{2}v^{\prime }.$$

We take appropriate linear combinations of equations (5.4)–(5.9) to replace *u*′ and *v*′ with $U^{\prime }$,

5.13 $$\begin{array}{rl} 0 & =ikU^{\prime }+Dw^{\prime },\;\text{on}\;0<z<2, \end{array}$$

5.14 $$\begin{array}{rl} \gamma \omega U^{\prime } & =-ik_{1}\bar{u}U^{\prime }-ik_{1}D\bar{u}w^{\prime }-\frac{2ik}{\prod Re}p^{\prime }+\frac{1}{Re}\left[-k^{2}+D^{2}\right]U^{\prime },\;\text{on}\;0<z<2, \end{array}$$

5.15 $$\begin{array}{rl} \gamma \omega w^{\prime } & =-ik_{1}\bar{u}w^{\prime }-\frac{2}{\prod Re}Dp^{\prime }+\frac{1}{Re}\left[-k^{2}+D^{2}\right]w^{\prime },\;\text{on}\;0<z<2, \end{array}$$

5.16 $$\begin{array}{rl} U^{\prime } & =0,\;w^{\prime }=0,\;p^{\prime }=0,\;\mathrm{at}\;z=2 \end{array}$$

5.17 $$\begin{array}{rl} \mathrm{and}\;\;kU^{\prime } & =-k_{1}LD\bar{u}r^{\prime },\;w^{\prime }=0,\;\mathrm{at}\;z=0. \end{array}$$

For *θ* < (*π*/2), which is relevant in the case of transverse bed-form structures such as canals, the Exner equation (5.10) does not always map perfectly onto the $U^{\prime }$ and *w*′ notation. We are left with a residual term involving *Du*′,

5.18 $$\omega r^{\prime }=-i\kappa \bar{F}DU^{\prime }-i\kappa k_{1}(Sd\bar{F}-\bar{F})Du^{\prime }\;\text{at}\;z=0.$$

In physical terms, the bed evolution in the Squire plane has two contributors: the first term on the right-hand side of (5.18) represents the bed evolution due to the flow field within the Squire plane. The second term represents the bed evolution due to the out-of-plane flow field, and hence it cannot be represented in terms of $U^{\prime }$ and *w*′. The out-of-plane contribution is a consequence of the nonlinearity of the sediment transport functional (2.9).

We derive an approximation for the residual term in (5.18) by assuming

5.19 θ≪1,

i.e. *k*_1_≪*k*_2_, which implies that the along-flow perturbations have very long wavelengths compared with the across-flow perturbations. As derived in appendix A, we write the Exner equation (5.10) in terms of $U^{\prime }$ and an additional term

5.20 $$\omega r^{\prime }=-ik\kappa \bar{F}DU^{\prime }+C(k,\theta )+O(\theta^{3}),\;\text{at}\;z=0,$$

where *C*(*k*, *θ*) is the quadratic correction factor for (5.18)

5.21 $$C(k,\theta )=-ik\theta \kappa V\bar{F}(Du^{\left(0\right)}(0)+\theta Du^{\left(1\right)}(0)).$$

The variable *V* represents

5.22 $$V=S\frac{d\bar{F}}{\bar{F}}-1,$$

and is non-negative, provided that the condition *m*≥1 is satisfied in (2.9).

Appendix A highlights that the choice of *θ* = 0 introduces a singular perturbation into (5.4)–(5.9), rendering the stability analysis inconclusive. Therefore, we perform the analysis by choosing a small non-zero value of *θ* satisfying (5.19). The quadratic expansion given in (5.21) is necessary to ensure that we solve the system of equations with consistent accuracy. Appendix A details the computations of *Du*^(0)^(0) and *Du*^(1)^(0). From this point onward, all variables are understood to be accurate up to second order in *θ*.

We introduce a streamfunction *ψ*(*z*) so that mass balance (5.13) holds implicitly

5.23 $$U^{\prime }=D\psi \;\mathrm{and}\;w^{\prime }=-ik\psi .$$

Streamfunction notation allows us to eliminate the pressure term and reformulate our equations into the Orr–Sommerfeld (OS) equation [63,64,66],

5.24 $$\gamma \omega \left[D^{2}-k^{2}\right]\psi =-ik\theta \left[\bar{u}D^{2}\psi -\psi D^{2}\bar{u}-k^{2}\bar{u}\psi \right]+\frac{1}{Re}{\left[D^{2}-k^{2}\right]}^{2}\psi ,\;\text{on}\;0<z<2.$$

The boundary equations arise from (5.16), (5.17) and (5.20),

5.25 $$\begin{array}{rl} & D\psi =0,\;\psi =0,\;\mathrm{at}z=2, \end{array}$$

5.26 $$\begin{array}{rl} & D\psi =-\sin(\theta )LD\bar{u}r^{\prime },\;\psi =0,\;\mathrm{at}\;z=0 \end{array}$$

5.27 $$\begin{array}{rl} \mathrm{and}\;\; & \omega r^{\prime }=-ik\kappa \bar{F}D^{2}\psi +C(k,\theta )\;\text{at}\;z=0. \end{array}$$

Given *k* and *θ*≪1, and setting a reference perturbation amplitude *r*′ = 1 for the bed-form, we compute *ω*_r_ by solving (5.24)–(5.27) for the unknowns *ψ* and *ω*.

### (b) Numerics

We reformulate the system of equations (5.24)–(5.27) as an eigenvalue problem and solve it numerically to obtain the eigenvalue–eigenvector pairs *ω* and (*ψ*, *r*′). To discretize the system of equations, we use a spectral Galerkin method originally proposed by Shen [67] and adapted for the current problem from [68,69]. We express the streamfunction as a linear combination of doubly integrated Legendre polynomials that vanish at the boundaries, plus two lower-order polynomials that incorporate the till–water boundary conditions. We present the details of the discretization in the electronic supplementary material.

The key benefit of Spectral Galerkin methods is that numerical accuracy does not depend on spatial discretization, but on the number of spectral elements. This method is particularly well suited to our physical system where spatial resolution of the near-bed dynamics, especially the computation of the derivatives *D*^2^*ψ* in (5.27), is crucial for determining bed stability. Spectral Galerkin methods are known to be highly accurate for solving the OS equation with homogenous boundary conditions [70], with the key advantage of not producing spurious eigenvalues.

## 6. Results

We study the stability properties of the meltwater film. We describe the system by the independent non-dimensional parameters *Re*, *L*, *α*, the perturbation angle *θ* and perturbation wavenumber *k*. We solve equations (5.24)–(5.27) to obtain the eigenvalues, *ω*, and corresponding eigenvectors (*ψ*, *r*′), where *ψ*(*z*) is the perturbed streamfunction, and *r*′ characterizes the amplitude of the bed-form perturbation. The eigenvalues are complex, i.e. *ω* = *ω*_r_ + *iω*_i_, with the sign of *ω*_r_ determining growth (*ω*_r_ > 0) or decay (*ω*_r_ < 0) of the perturbations in time.

The primary motivation for our analysis here is to understand the physical processes leading to the initiation of efficient drainage in the along-flow direction such as till-incised canals. We hence consider perturbations that are close to perpendicular to the main flow direction, *θ*≪1, and evaluate which perturbations grow the fastest.

### (a) System instability

**Figure 4.**
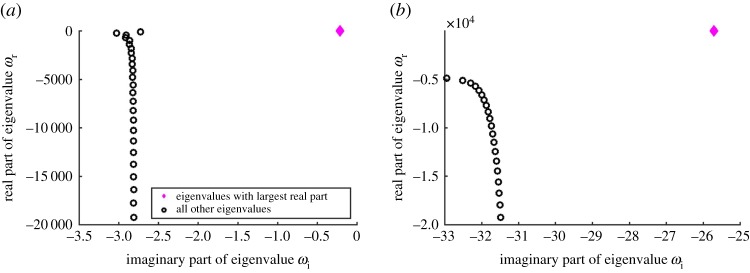
Plot of the eigenspectra for (*a*) *k* = 1 and (*b*) *k* = 20. The panels show the first 20–25 eigenvalues with the largest real parts. *θ* = 0.01, *Re* = 20, *L* = 10^−3^, *α* = 10^−3^. (Online version in colour.)

For demonstration purposes, we consider a meltwater film with the following values for the independent non-dimensional parameters: Reynolds number *Re* = 20, grain-to-film size ratio *L* = 10^−3^ and surface slope *α* = 10^−3^. This system corresponds to film thickness 2*H* ≈ 4 mm and grain diameter *D* ≈ 2 μm (clay-like). We set *θ* = 0.01 to consider perturbations that are near-perpendicular to the main flow direction. We discretize the system with the number of spectral elements *N* = 300 and plot the spectra of eigenvalues in figure 4*a*,*b* for wavenumbers *k* = 1 and *k* = 20, respectively.

Figure 4 highlights that the eigenvalue with the largest real part stands out from the cluster of the other eigenvalues of the system. The distinctness of this particular eigenvalue suggests that the corresponding solution (mode) represents a set of physical processes that are different from the other modes of the system.

**Figure 5.**
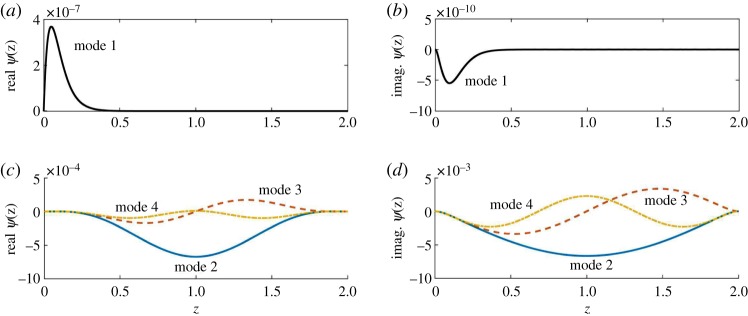
Eigenfunctions *ψ*(*z*) corresponding to the top four eigenvalues with the largest real parts. Panels (*a*,*b*) indicate that the eigenfunction corresponding to the first mode has a localized structure near the bed (*z* = 0). The other modes appear symmetric and span the thickness of the film. *k* = 20, *θ* = 0.01, *Re* = 20, *L* = 10^−3^, *α* = 10^−3^. The structure of the first mode becomes more localized as *k* increases. The large value of *k* is chosen to visually highlight the localized structure. The first mode *ψ* (*a*) real part and (*b*) imaginary part. (*c*) The next three modes (real parts). (*d*) The next three modes (imaginary parts). (Online version in colour.)

To better understand the underlying processes, we plot the eigenfunctions *ψ*(*z*) corresponding to the respective eigenvalues *ω* in figure 5. The first eigenfunction, corresponding to the eigenvalue with the largest real part, has a structure localized in the region close to the bed (*z* = 0), which is indicative of near-bed processes. This particular eigenpair (*ψ*, *ω*) corresponds to the mode dominated by bed evolution processes. All the other eigenfunctions plotted in figure 5*c*,*d* are characterized by symmetric profiles spanning the entire domain. They represent hydrodynamic responses to the perturbation which arise even in the absence of a dynamic bed [66]. From this point onward, we denote the eigenpair (*ψ*, *ω*) with the near-bed localized structure as the sediment transport eigenpair.

**Figure 6.**
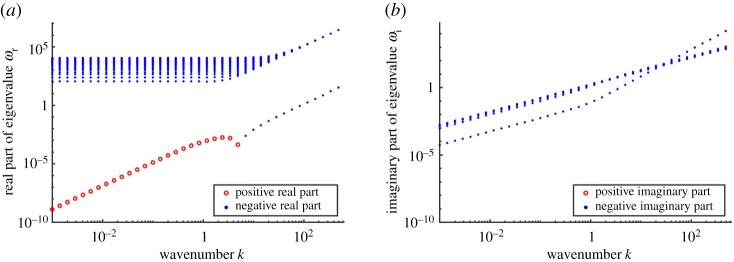
Plot of the eigenspectra over a range of *k* for the top 20 eigenvalues with the largest real parts. *θ* = 0.01, *Re* = 20, *L* = 10^−3^, *α* = 10^−3^. The eigenvalue that visually stands out in magnitude for each *k* corresponds to the sediment transport eigenvalue. (*a*) Real part of eigenvalue and (*b*) imaginary part of eigenvalue. (Online version in colour.)

Figure 6 shows the eigenspectra for a range of wavenumbers 10^−3^ ≤ *k* ≤ 10^3^. For each wavenumber *k* on the *x*-axis, we plot on the *y*-axis the top 20 eigenvalues with the largest real parts. Most of the eigenvalues in the spectra lie in the upper half of figure 6*a* and have negative real parts. For these eigenvalues, we compute the corresponding eigenfunctions and confirm that they have profiles similar to those in figure 5*c*,*d*, which indicates that they are hydrodynamic modes. The negative real parts for the hydrodynamic eigenvalues show that, under the given parameter regime, there is no turbulence in the film [66].

The eigenvalue with the largest real part stands out for each value of *k*. Its eigenfunction profile confirms that it is the sediment transport eigenvalue. Figure 6*a* further indicates that for small *k*, the sediment transport eigenvalue satisfies *ω*_r_ > 0, indicating a morphological instability.

This instability is governed by the bed-load transport patterns that arise from the near-bed flow profile of the film. To study the processes that shape the near-bed flow, we distinguish three sub-processes within the hydrodynamics of the system: acceleration, advection and diffusion

6.1 $$\gamma \overset{\text{acceleration}}{\overbrace{\omega \left[D^{2}-k^{2}\right]\psi }}=\overset{\text{advection}}{\overbrace{-ik_{1}\left[\bar{u}D^{2}-D^{2}\bar{u}-k^{2}\bar{u}\right]\psi }}+\frac{1}{Re}\overset{\text{diffusion}}{\overbrace{{\left[D^{2}-k^{2}\right]}^{2}\psi }}.$$

Our model resolves the above-mentioned hydrodynamic sub-processes within the Squire plane only. For near-transverse perturbations (*θ*≪1), the instability is not only affected by the processes within the Squire plane but also the flow-field outside the plane. More specifically, the Exner equation (5.27) includes the correction term *C*(*k*, *θ*), which represents the out-of-plane contribution to the instability.

In the upcoming subsections, we investigate how the three hydrodynamic sub-processes couple with bed-load transport to affect the stability of the system at different wavenumbers *k*. We also isolate and highlight the influence of the out-of-plane dynamics in these investigations.

### (b) Viscous diffusion causes bed-form migration

**Figure 7.**
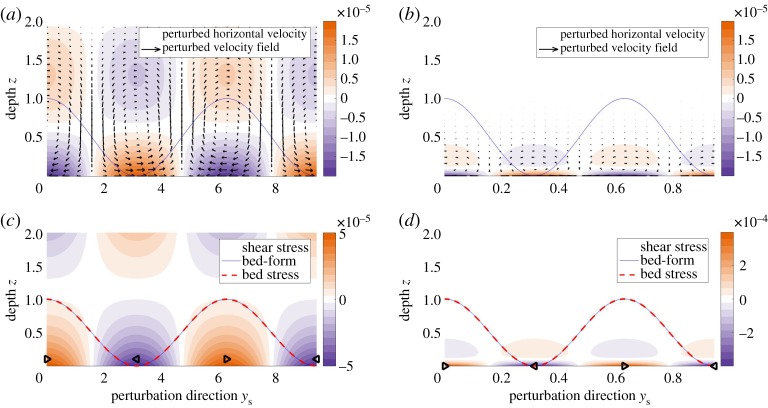
The diffusion-only regime. The bed-form (solid blue line) and bed stress (dashed red line) are in phase. *Re* = 10^−6^, *L* = 10^−3^, *α* = 10^−3^, *θ* = 0.01. Most of the steady-state flow comes out of the page. (*a*) The velocity field and the colour plot of Squire horizontal velocity U for the long wavelength regime (*k* = 1). (*c*) The shear stress τ in the Squire direction for *k* = 1. The triangle markers indicate the sign of the stress (right-ward pointing stands for positive values). Panels (*b*) and (*d*) represent the short wavelength regime (*k* = 10). The solid line represents the bed-form perturbation. Its amplitude is not to scale. Velocities (*a*) *k* = 1 and (*b*) *k* = 10. Shear stress (*c*) *k* = 1 and (*d*) *k* = 10. (Online version in colour.)

We first study a diffusion-only system. To suppress the advection and acceleration terms, we consider the regime *Re*≪1. We numerically solve the system of equations (5.24)–(5.27), and we isolate the sediment transport eigenvalue *ω* and the corresponding streamfunction *ψ*. Figure 7 shows the velocity and shear stress perturbations for the sediment transport eigenpair (*ω*, *ψ*). Panels (*a*,*c*) consider the case *k* = 1 and show the Squire velocity vector field (U,w), as well as the colour plots of U and the shear stress

6.2 $$\tau =D^{2}\psi .$$

Panels (*b*,*d*) represent the case of perturbations with short wavelengths (*k* = 10).

The horizontal velocities at the till–water interface are generated by the perturbations in the bed, as seen in (5.26). The velocities then propagate toward the ice as a result of viscous diffusion, and their corresponding gradients create the stress field. The near-bed vertical velocities and associated circulation cells are generated by mass conservation over the horizontal velocity gradients at the boundary.

Figure 7 shows that bed-stress and bed-form are in phase, in which case (5.27) indicates that the real part of the sediment transport eigenvalue is zero. Since the system perturbations have the form $\exp(k_{1}x+k_{2}y+\omega t)$, the equation *ω*_r_ = 0 implies that the bed-form neither amplifies nor decays, but simply migrates along the bed. We conclude that diffusion by itself does not affect the stability of the system. In other words, a diffusion-only system is neutrally stable.

We develop a reduced model to better understand the physics in the diffusion-only case that leads to *ω*_r_ = 0. Figure 7*b* suggests the formation of a boundary layer for the short wavelength regime (*k*≫1). We set perturbation amplitude *r*′ = 1. We re-scale the variables

6.3 $$z^{⋆}=kz,\;\psi^{⋆}=\frac{k\psi }{L\sin\theta },\;\omega^{⋆}=\frac{\omega }{k^{2}\bar{F}\kappa L\sin\theta },\;C^{⋆}(k,\theta )=\frac{C(k,\theta )}{k^{2}\bar{F}\kappa L\sin\theta }.$$

The re-scaled OS equation (5.24) takes the form

6.4 $$a_{1}\omega (D^{2}-1)\psi^{⋆}=-ia_{2}\left[\left(2z^{⋆}-k^{-1}{z^{⋆}}^{2}\right)(D^{2}-1)+2k^{-1}\right]\psi^{⋆}+{\left(D^{2}-1\right)}^{2}\psi^{⋆},$$

6.5 $$\text{where}\;a_{1}=\bar{F}L\gamma Re\kappa \sin\theta ,\;a_{2}=Rek^{-2}\sin\theta .$$

The limits *a*_1_ → 0 and *a*_2_ → 0 suppress the acceleration and advection terms. We may interpret this regime as a highly viscous flow, *Re* → 0. We perform an asymptotic expansion of the variables to study the short wavelength regime

6.6 $$z^{⋆}=z^{\left(0\right)}+O\left(\frac{1}{k}\right),\;\psi^{⋆}=\psi^{\left(0\right)}+O\left(\frac{1}{k}\right),\;\omega^{⋆}=\omega^{\left(0\right)}+O\left(\frac{1}{k}\right)\;\mathrm{and}\;C^{⋆}=-2iV+O\left(\frac{1}{k}\right),$$

where the asymptotic behaviour of *C* is provided in appendix A.

We reduce equations (6d), (5.26), (5.25) and (5.27) to leading order as *k* → ∞,

6.7 $$\begin{array}{rl} 0 & ={\left(D^{2}-1\right)}^{2}\psi^{\left(0\right)},\;\text{on}\;0<z^{\left(0\right)}<\infty , \end{array}$$

6.8 $$\begin{array}{rl} D\psi^{\left(0\right)} & =-2,\;\psi^{\left(0\right)}=0,\;\text{at}\;z^{\left(0\right)}=0, \end{array}$$

6.9 $$\begin{array}{rl} D\psi^{\left(0\right)} & \to 0,\;\psi^{\left(0\right)}\to 0,\;\text{as}\;z^{\left(0\right)}\to \infty \end{array}$$

6.10 $$\begin{array}{rl} \mathrm{and}\;\;\omega^{\left(0\right)} & =-iD^{2}\psi^{\left(0\right)}-2iV\;\text{at}\;z^{\left(0\right)}=0. \end{array}$$

The leading-order solution of the reduced boundary layer model is given by

6.11 $$\psi^{\left(0\right)}=-2z^{\left(0\right)}\exp(-z^{\left(0\right)})\;\mathrm{and}\;\omega^{\left(0\right)}=-4i-2iV,$$

where the term −4i arises as a result of the coupling between diffusion and sediment transport in the Squire plane. Since it is pure imaginary, the above analysis supports the hypothesis that diffusion in the Squire plane is neutrally stable in the short wavelength regime, i.e. diffusion does not affect the stability of the system. The second term in (6.11), −2i*V* , is the out-of-plane contribution to the stability. It is also pure imaginary, suggesting that the out-of-plane flow field does not affect the stability of the system at short wavelengths either.

In the long wavelength regime (*k*≪1), we consider the following re-scaled variables:

6.12 $$\psi^{⋆}=\frac{\psi }{L\sin\theta },\;\omega^{⋆}=\frac{\omega }{\bar{F}Lk\kappa \sin\theta },\;C^{⋆}(k,\theta )=\frac{C(k,\theta )}{k\bar{F}\kappa L\sin\theta },$$

to obtain the re-scaled OS equation

6.13 $$b_{1}k\omega^{⋆}(D^{2}-k^{2})\psi^{⋆}=-ib_{2}k\left[\left(2z-z^{2}\right)(D^{2}-k^{2})+2\right]\psi^{⋆}+{\left(D^{2}-1\right)}^{2}\psi^{⋆},$$

6.14 $$\text{where}\;b_{1}=\bar{F}L\gamma Re\kappa \sin\theta ,\;b_{2}=Re\sin\theta .$$

The advection and acceleration terms automatically vanish as *k* → 0. We consider the asymptotic expansion of the system at *k* = 0

6.15 $$z=z^{\left(0\right)}+O(k),\;\psi^{⋆}=\psi^{\left(0\right)}+O(k),\;\omega^{⋆}=\omega^{\left(0\right)}+O(k),\;C^{⋆}=-iV+O(k).$$

To leading order, equations (5.24)–(5.27) reduce to

6.16 $$\begin{array}{rl} & 0=D^{4}\psi^{\left(0\right)},\;\text{on}\;0<z<2, \end{array}$$

6.17 $$\begin{array}{rl} & D\psi^{\left(0\right)}=-2,\;\psi^{\left(0\right)}=0,\;\text{at}\;z^{\left(0\right)}=0, \end{array}$$

6.18 $$\begin{array}{rl} & D\psi^{\left(0\right)}=0,\;\psi^{\left(0\right)}=0,\;\text{as}\;z^{\left(0\right)}=2 \end{array}$$

6.19 $$\begin{array}{rl} \mathrm{and}\;\; & \omega^{\left(0\right)}=-iD^{2}\psi^{\left(0\right)}-iV\;\text{at}\;z^{\left(0\right)}=0, \end{array}$$

as *k* → 0. Omitting the asymptotic notation, the leading-order solution is given by

6.20 $$\psi =-2z+2z^{2}-\frac{1}{2}z^{3},\;\omega =-4i-iV,$$

and shows that *ω*_r_ = 0 as *k* → 0. As in the short-wavelength case, both the terms constituting *ω* in (6.20), −4i, corresponding to the contribution of the viscous diffusion within the plane, and the term −i*V* , corresponding to the effect of the out-of-plane flow field, have zero real parts. The solution above implies that the diffusion-only system is neutrally stable.

### (c) Advection destabilizes the system

**Figure 8.**
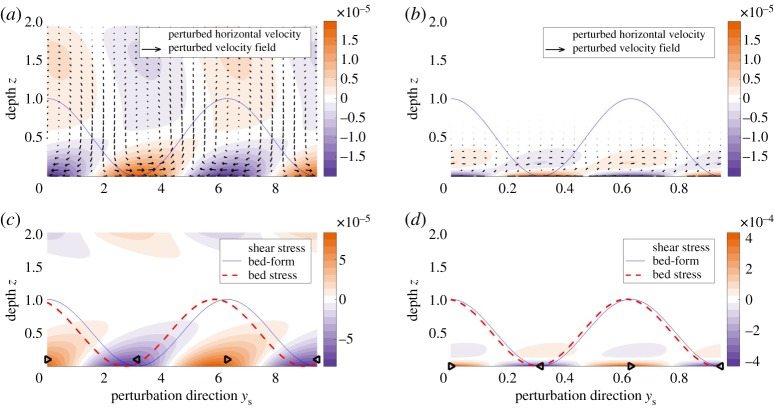
The advection–diffusion regime. Advection creates a right-ward skew of velocity and phase advance of the shear stress. *Re* = 500, *L* = 10^−4^, *α* = 10^−4^, *θ* = 0.1. Most of the steady-state flow comes out of the page. The plot set-up is identical to figure 7. The choice of somewhat larger *θ* allows us to visually distinguish the phase difference between bed-form and bed-stress. Velocities (*a*) *k* = 1 and (*b*) *k* = 10. Shear stress (*c*) *k* = 1 and (*d*) *k* = 10. (Online version in colour.)

We study the advection–diffusion system and identify the effect of advection on the film instability. The acceleration term is suppressed under the condition *γ*≪1. Equation (3.7) suggests that this condition is achieved with *L*≪1. Figure 8 plots the velocity and shear stress for the advection–diffusion regime. The computed velocity field exhibits a right-ward skew. This skew causes a left-ward phase shift of the shear stress, especially the bed stress, resulting in a phase advance over the bed-form. The bed stress advancing ahead of the bed-form implies that Im(τ) > 0, which yields *ω*_r_ > 0 from (5.27) and (6.2), suggesting that advection contributes to the instability of the system.

We use a reduced model to test our hypothesis that the advection–diffusion force balance leads to *ω*_r_ > 0 in the short wavelength regime. We take the limit *a*_1_ → 0 of the re-scaled OS equation (6d) to suppress the acceleration term. The short wavelength asymptotic expansion (6.6) yields

6.21 $$2ia_{2}z^{\left(0\right)}(D^{2}-1)\psi^{\left(0\right)}={\left(D^{2}-1\right)}^{2}\psi^{\left(0\right)},\;\text{on}\;0<z^{\left(0\right)}<\infty ,$$

while the boundary equations are given by (6.8)–(6.10). The leading-order solution is given by

6.22 $$\psi^{\left(0\right)}=\frac{2\int_{0}^{z}\int_{v}^{\infty }e^{2v-s-z}\mathrm{Ai}(c^{-1}s+c^{2})\;dsdv}{\int_{0}^{\infty }e^{-s}\mathrm{Ai}(c^{-1}s+c^{2})\;ds},\;\omega^{\left(0\right)}=\frac{-2i\mathrm{Ai}(c^{2})}{\int_{0}^{\infty }\;e^{-s}\mathrm{Ai}\left(c^{-1}s+c^{2}\right)ds}-2iV,$$

where $c=(1/\sqrt[{3}]{2ia_{2}})$ with arg(*c*) =  − (*π*/6), and Ai(*s*) is one of the two standard linearly independent solutions of the system *D*^2^*f* = *sf*. The integrals in (6.22) converge as a consequence of the exponential decay rate of Ai(*s*) for −(∏/3)<arg⁡(s)<(∏/3).

The electronic supplementary material, figure S2, plots *ω*^(0)^ over a range of the simplified parameter *a*_1_. The figure shows that *ω*^(0)^_r_ > 0, and thus supports our hypothesis that the interplay of advection and diffusion causes the instability.

The asymptotic analysis from the previous section suggests that diffusion dominates in the long-wavelength regime, yielding a neutrally stable system, i.e. *ω*_r_ = 0. Assuming that the sediment transport eigenvalue varies continuously with the wavenumber *k*, if *ω*_r_ is positive at wavenumber *k*_0_, then it is likely that *ω*_r_ decays to 0 while maintaining its sign over the range 0 < *k* ≤ *k*_0_. This hypothesis appears to hold in figure 6, where *ω*_r_ peaks at *k* = 3 and diminishes to 0 as *k* becomes small.

**Figure 9.**
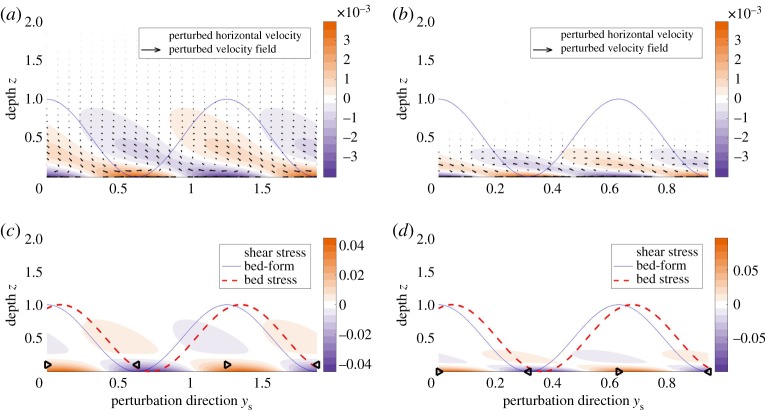
Short-wavelength regime. Acceleration creates a left-ward skew of velocity and phase lag of the bed stress. *Re* = 1, *L* = 0.02, *α* = 0.02, *θ* = 0.1. Most of the steady-state flow comes out of the page. The plot set-up is identical to figure 7. The choices of somewhat larger *L*, *α* and *θ* allow us to visually distinguish the phase difference between bed-form and bed-stress. Velocities (*a*) *k* = 5 and (*b*) *k* = 10. Shear stress (*c*) *k* = 5 and (*d*) *k* = 10. (Online version in colour.)

We sketch the physics of destabilization via advection in figure 9*a*. The component of steady-state velocity $\bar{U}$ advects momentum to the perturbed velocity field, causing the rightward skew that leads to a phase advance in the bed stress, which amplifies the bed-form perturbations.

### (d) Fluid acceleration stabilizes the system for short wavelengths

The computed spectra in figure 6*a* show that the real part of the sediment transport eigenvalue becomes negative as the wavenumber becomes large, indicating that the film suppresses short-wavelength structures at the bed. Since our analysis suggest that advection is a destabilizer and diffusion does not influence stability, we study the role of acceleration as a potential stabilizer.

**Figure 10.**
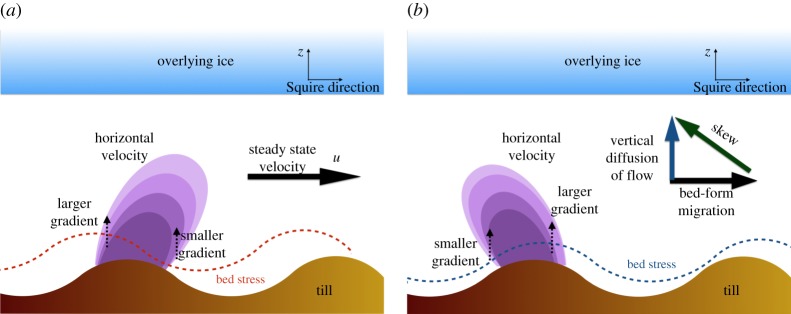
Representations of the physics for the stabilization and destabilization of the meltwater film caused by acceleration and advection, respectively. Skews in the velocity field leads to phase shifts in the bed stress, which affect the stability of the system. (*a*) Advection destabilizes and (*b*) acceleration stabilizes. (Online version in colour.)

We consider the system in the short-wavelength regime (*k*≫1). Figure 10 shows the plots of computed velocity fields and shear stresses for this parameter regime. In contrast to the right-ward velocity skew in the advection–diffusion case, figure 10*a* highlights a left-ward velocity skew, which creates a right-ward phase shift of the bed stress. The bed stress lagging behind the bed-form implies that Im(τ) < 0, and hence yields *ω*_r_ < 0. We conclude that acceleration contributes to the short-wavelength stabilization of the system.

We study the nature of the stabilization for the short wavelength regime with a reduced model. We follow the asymptotic analysis outlined in (6.3)–(6.6). The limit *k* → ∞ of the reduced OS equation (6d) removes all the advection terms

6.23 $$a_{1}\omega {\left(D^{2}-1\right)}^{2}\psi^{\left(0\right)}={\left(D^{2}-1\right)}^{2}\psi^{\left(0\right)},\;\text{on}\;0<z<\infty .$$

The leading-order solution of (6.23) and (6.8)–(6.10) is given by

6.24 $$\psi^{\left(0\right)}=\frac{2\left[e^{-z^{\left(0\right)}}-e^{-z^{\left(0\right)}\sqrt{1+a_{1}\omega }}\right]}{1-\sqrt{1+a_{1}\omega^{\left(0\right)}}},\;\omega^{\left(0\right)}=-2a_{1}+2i-2iV+2i\sqrt{{\left(1+a_{1}i\right)}^{2}-2a_{1}iV},$$

where all square roots are taken with non-negative imaginary parts (see the electronic supplementary material for more details). The out-of-plane flow-field is coupled to the in-plane solution, unlike in the previous cases of advection and diffusion. Since the square root in (6.24) has a non-negative imaginary part, the eigenvalue has a negative real part, i.e. *ω*_r_ < 0. The real part of the eigenvalue remains negative even if we ignore the out-of-plane contribution *C*(*k*, *θ*), i.e. *V* = 0. Our analysis thus suggests that fluid acceleration stabilizes short-wavelength perturbations.

We summarize the physical intuition for the stabilizing effect of acceleration in figure 9*b*. The key insight, shown in figure 6*b*, is that the bed-form migration speed scales with the wavenumber (*ω*_i_∼*k*). As *k* increases, the speed of bed-form migration becomes comparable to the rate of momentum diffusion due to fluid viscosity. The relative motion between the fluid velocities that diffuse toward the ice and the bed-form migration results in a left-skew of the velocity. The skewed velocity field creates a corresponding phase lag in the bed stress that stabilizes short-wavelength perturbations in the system.

### (e) The most unstable perturbation wavelength

We define the wavenumber *k*_*u*_ as the one corresponding to the sediment transport eigenvalue with the largest positive real part. Since perturbations at this wavenumber grow at the fastest rate, the corresponding wavelength *λ*_*u*_ = 2*πk*^−1^_*u*_ is indicative of the initial spacing for the bed heterogeneity generated by the morphological instability described in this paper. We perform a study for how *λ*_*u*_ varies over the three independent non-dimensional parameters: *Re*, the Reynolds number; *L*, the grain-to-film size ratio, and *α*, the surface slope. We set *θ* = 0.01, which implies that most of the perturbation is across-flow.

Since the model does not resolve the long-term evolution of the emerging spatial heterogeneity at the bed, it is unlikely that *λ*_*u*_ matches field observations of spacing between canals or other evolved structures. However, potential laboratory experiments may provide a means to validate our model results.

**Figure 11.**
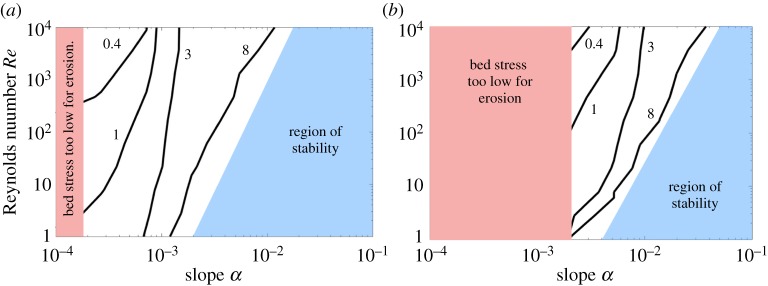
Contours of unstable wavelength *λ*_*u*_ over parameters *Re* and *α*. Solid line contours and their neighbouring numbers represent values of *λ*_*u*_. The red-shaded rectangular region indicates that the bed stress is insufficient to erode sediment. The shaded trapezoidal blue region highlights a region of stability. Panels (*a*) and (*b*) represent *L* = 10^−3^ and *L* = 10^−2^, respectively. (Online version in colour.)

Figure 11 shows the results of the sensitivity analysis. The shaded rectangular region on the left represents the regime where (3.10) is not satisfied, indicating that the bed stress is insufficient to erode the sediment. The stress threshold scales with grain size (∼*L*) as it becomes increasingly difficult for the fluid to erode larger grains (figure 2*b*).

The contour values of *λ*_*u*_, shown in figure 11, suggest that the most unstable wavelength is within one order of magnitude of the film thickness itself. The value of *λ*_*u*_ generally decreases with *Re* and increases with *α*. The region of stability shrinks as *L* increases.

## 7. Discussion

In §6, we identified an instability where a meltwater film grows unstably by carving into the sediment layer beneath. Our main motivation is to understand the initiation of efficient drainage elements for which the main axes are approximately aligned with the main flow direction, as is the case for canals incised into the till [32,33]. This kind of drainage element would emerge as out-of-bed perturbations that are near-transverse to the main flow direction. We show that these perturbations are indeed unstable, and that this instability occurs prior to the onset of turbulence.

Our analysis indicates that the physics of the bed instability is similar to that of granular ripple formation [40,43]. However, the bed structure that emerges from the instability we discuss is distinct from ripples. It is near-transverse to the main flow direction, whereas ripples constitute bed perturbations in the along-flow direction. Although we do not explicitly study the case of ripples, we expect a meltwater film on soft erodible till to exhibit both ripples [44] as well as near-transverse variability, resulting in a spatially heterogeneous drainage structure. The emerging bed-form may eventually evolve into efficient drainage elements such as till-incised canals [32,33], but the long-term evolution of the system is beyond the scope of our linearized stability analysis.

### (a) Hydrodynamics induces short-wavelength stabilization of films

Morphological instabilities of flow over erodible beds are classical topics in fluid dynamics and hydrology as reviewed, for example, by [40,43]. Kennedy [37,43] was among the first to explain the dynamics of the granular ripple instability. He identified that the instability arises from the phase advance of the bed shear stress over the bed-form, which is a result of near-bed flow advection being countered by the shear stress at the bed. However, the potential flow model used by Kennedy [37] aligned the bed-form and the bed stress exactly, thus requiring an externally imposed phase advance to activate the ripple instability. Shallow water models predicted stability of the bed at all wavelengths since they could not resolve the differences between mean flow and near-bed flow [42]. Rotational flow models which resolve the vertical flow velocities, e.g. [38,39], addressed the phase advance problem successfully, and we follow this modelling approach to understand the evolution of meltwater films. Results from previous rotational flow models [38,39,41,44] are consistent with the advection-induced instability mechanism discussed by Kennedy [37,43] and presented in this paper (figure 9*a*).

The theory of flow over erodible beds was originally intended for analyses of granular ripples on beaches and riverbeds [40]. Therefore, most film models assume a free surface boundary at the top [38,39,41]. Since our model represents meltwater films capped by ice, i.e. a fixed lid boundary condition, it does not exhibit the stabilizing effect of a free surface at subcritical flow [40], nor does it prompt the formation of antidunes at supercritical flow [41,44]. The lack of stabilization from a free surface suggests that alternate mechanisms operate to stabilize films with fixed lids.

The direction of the bed perturbation presents another difference between our study and others. We focus on bed structures near-perpendicular to the main flow because we are interested in the initiation of canals with axes approximately aligned with the film flow direction. Most previous studies [37–39], by contrast, analyse ripple formation along the film flow direction. Our results show that bed structures near-perpendicular to the main flow are unstable. However, the study by Devauchelle *et al.* [44], which includes oblique perturbations as well as a fixed lid boundary condition, reports that the film stabilizes bed structures that are near-perpendicular to the main flow. This disagreement between our findings and [44] stems from the differences between the respective mechanisms of bed-form stabilization within the underlying models.

As discussed in the review by Charru *et al.* [40], most models of flow over erodible beds introduce two sediment-based mechanisms to add stability to the system: a saturation lag in the bed-load density, which imposes a minimum bed-form wavelength, and a gravity effect, where an uneven bed tends to flatten itself diffusively due to grain motion along small-scale bed slopes. Devauchelle *et al.* [44] obtain stability for short wavelengths as well as for near-perpendicular perturbations as a result of these two mechanisms. The saturation lag mechanism is supported by experimental [71] and observational evidence [72] in the case of aeolian dunes, but it is not clear how effective it would be for a non-turbulent thin meltwater film where grain saltation is suppressed. The gravity effect is based on experimental studies of grain incipient motion for flow over an inclined bed, e.g. [73,74]. This effect is most pronounced when the system is near the threshold Shields stress, and it vanishes as the bed stress becomes large [40]. The strength of the gravity effect is a source of uncertainty since there is no comprehensive study on how it varies with grain properties such as diameter, density, shape and cohesion. The uncertainty is magnified in the case of subglacial sediments, for which observational records are sparse and varied.

Our model invokes neither of these sediment-based stabilization mechanisms. Instead, we show the hydrodynamics itself stabilizes short wavelengths through the acceleration–diffusion mechanism outlined in figure 9*b*. The stabilizing feedback arises from resolving the linear time-evolution response of the hydrology to the perturbation. Figure 9*b* shows that the bed stress lags the bed-form when the rate of momentum diffusion via viscosity is comparable to the bed-form migration speed. Previous models [38,39,41,44] are unable to reproduce this phase lag, because they assume quasi-steady flow, namely that the fluid flow adapts instantaneously to any changes in the bed. Quasi-steady flow is justified by arguing that hydrology operates significantly faster than sediment transport (*γ*≪1). While the assumption may be true for the mean flow of the film, the separation of time scales is unlikely to hold in the vicinity of the bed. Our results from figure 6 show that even a three-order magnitude difference (*γ*∼10^−3^) maintains the acceleration-based stabilization effect at wavelengths around 0.1 times the film size.

In a real meltwater film setting, both the hydrology-based mechanism and the sediment-based mechanisms likely contribute to the stability of the system. The latter, however, appear to manifest only in specific regimes such as in the presence of saltating flow or, in case of the gravity effect, when the system is close to the critical Shields threshold [40]. Nevertheless, it is possible that the sediment-based mechanisms stabilize near-perpendicular perturbations in meltwater films and limit the instability to the formation of oblique drainage elements such as bars [44].

### (b) Drainage elements on soft beds versus hard beds

Walder & Fowler [32] and Ng [33] suggest that efficient drainage systems on soft subglacial sediment beds take the form of canals that are incised into the till. Canals are commonly observed in the subglacial setting (e.g. Rutford Ice Stream, West Antarctica [35]), but it is unclear which processes lead to their formation. If thin meltwater films collapse by carving into the ice as a consequence of Walder's instability [19,22], Röthlisberger channels [24] will dominate the early evolution of the hydrological system. This study presents an alternate framework where meltwater films on soft beds develop spatially heterogeneous drainage elements by eroding the till beneath. We suggest that the emerging heterogeneity at the bed may eventually lead to the formation of till-incised canals, but explicitly studying this evolution is beyond the scope of our linearized stability analysis.

Subglacial drainage systems with a dynamic till have been studied previously, but the initiation of such systems has not been addressed directly. Ng [33] describes the coupled dynamics of hydrology and till in fully developed subglacial canals. He presents equilibrium conditions of a till-incised canal system that spans tens of kilometres. At this length scale, canal dynamics is dictated by large-scale mass fluxes of water and sediment rather than smaller scale features such as bed geometry and vertical flow profiles. Our model provides a complementary approach in the sense that we study meltwater films at the length scale of the film thickness and resolve bed geometry and near-bed flow dynamics. By studying canals in the context of an overarching spatially heterogeneous drainage system, it may be possible to alleviate some of the difficulties in defining meaningful physical conditions that define the edges of the canal [33].

Kyrke-Smith & Fowler [21] develop a model to understand the evolution of meltwater films on soft beds, where they include the processes of till erosion and deformation and meltwater generation. Since Walder's mechanism of film expansion via dissipation is known to make meltwater films unstable, [21] introduces the framework of supporting clasts, developed by Creyts & Schoof [23], to suppress Walder's instability. The key insight from Creyts & Schoof [23] is that clasts distributed within the till bear the majority of the ice overburden stress, and this stress localization leads to faster closure of the ice roof, thus adding stability to the film.

While the framework of supporting clasts is well-suited to subglacial water systems over hard beds, it is not clear that the framework translates to plastic beds. The failing basal till underneath ice streams [9,10,31] is unlikely to support clasts in the same way as a hard bed. Our study mimics the set up of Walder's instability [22], where a thin film of meltwater separates the ice and the soft sediment. While the assumption of protruding clasts introduces static roughness to the bed, the instability described in this study leads to dynamic roughness in the form of emerging spatial heterogeneity at the bed. This dynamic roughness, at least initially, is purely a consequence of the coupled dynamics of hydrology and sediment transport: the water fluxes are far too low to introduce any Walder-type interaction with the ice.

Our study highlights a contrast to the condition of stability that arises from the supporting clasts framework of Creyts & Schoof [23]. For hard beds, Creyts & Schoof argue that the largest clast size controls the onset of instability within the meltwater film, since the ice overburden stress localizes over the largest clasts as the film grows. For soft erodible beds, our study suggests that sediment grains with the smallest size control the onset of instability, since they are the easiest to erode. While our study does not directly account for multiple grain sizes, work by [75] suggests the possibility that film flow can channelize by preferentially eroding smaller-sized grains. Based on our findings, we hypothesize physical differences between hard and soft beds, where the smallest grain size controls stability for soft beds, unlike in the hard-bed case where stability is controlled by the largest clast size.

## 8. Conclusion

The linearized stability analysis in this paper highlights that water transport over soft beds is associated with dynamic bed-form evolution in the subglacial till. The analysis elucidates the conditions under which a spatially heterogeneous drainage structure carved into the sediment can emerge from a flat bed as a result of a morphological instability. Our theory would be testable against idealized laboratory experiments of thin film flow over granular beds in a Hele–Shaw cell, which, to our knowledge, are not currently available. Comparing the fastest growing wavelengths of this instability to observations of subglacial morphologies in the field [76] would require follow-up work that captures the nonlinear evolution of the incipient bed-form into its fully fledged form, for example, through a depth-resolved, direct numerical simulation.

## Supplementary Material

Mathematical Derivations

## Figures and Tables

**Figure 12.**
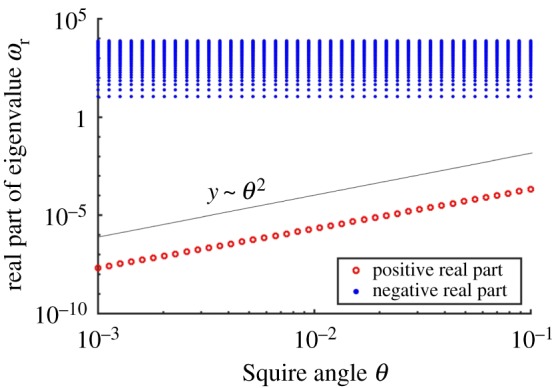
Numerical solution for the sediment transport eigenvalue $\tilde{\omega }$ varies as *O*(*θ*^2^). The hydrodynamic eigenvalues are independent of *θ*. *k* = 1, *Re* = 20, *L* = 10^−3^, *α* = 10^−3^. (Online version in colour.)

## Data accessibility

The source code for the numerical solutions can be accessed at http://zapad.stanford.edu/ineel/meltwater-film-stability.

## Appendix A. Small angle approximation

The system of equations that describe our model of meltwater film stability is given by (5.24)–(5.26), along with the exact linearized Exner equation (5.18) in streamfunction notation (5.23)

A 1 $$\omega r^{\prime }=-i\kappa \bar{F}kD^{2}\psi -i\kappa k_{1}(Sd\bar{F}-\bar{F})Du^{\prime }\;\text{at}\;z=0.$$

The Orr–Sommerfeld solver described in §5c cannot resolve the term *Du*′(0) since that term represents the contribution of the flow field outside the Squire plane to the bed-form evolution within the Squire plane. Instead, our approach is to approximate *Du*′(0) for small *θ* (5.19). We perform a quadratic expansion around *θ* = 0,

A 2 $$\omega =\omega^{\left(0\right)}+\theta \omega^{\left(1\right)}+\theta^{2}\omega^{\left(2\right)}+O(\theta^{3})$$

and

A 3 $$f(z)=f^{\left(0\right)}(z)+\theta f^{\left(1\right)}(z)+\theta^{2}f^{\left(2\right)}(z)+O(\theta^{3}),$$

where *f*(*z*) stands for a generic variable *u*′, *v*′, *w*′, *p*′, $U^{\prime }$, *ψ*.

The need for second-order accuracy in *θ* is suggested by figure 12. It shows that the numerical solution (denoted by tilde) for the sediment transport eigenvalue $\tilde{\omega }$ of (5.24)–(5.27) satisfies $\tilde{\omega }∼\theta^{2}$. Under a quadratic expansion, we are guaranteed that $\omega -\tilde{\omega }=O(\theta^{3})$, where *ω* is the exact solution for the system (5.24)–(5.26), (A 1), ensuring that $\tilde{\omega }$ is a sufficiently accurate approximation of *ω*.

To ensure that the error is at most *O*(*θ*^3^), we replace *Du*′ in (A 1) with *Du*^(0)^ + *θDu*^(1)^,

A 4 $$\omega r^{\prime }=-i\kappa \bar{F}D^{2}\psi +C(k,\theta )+O(\theta^{3}),$$

where $V=(Sd\bar{F}/\bar{F})-1$, and *C*(*k*, *θ*) is the correction term given by (5.21),

$$C(k,\theta )=-ik\theta \kappa V\bar{F}(Du^{\left(0\right)}+\theta Du^{\left(1\right)}).$$

Since the difference between the exact Exner equation (A 1) and the approximated version (A 4) is an *O*(*θ*^3^) constant, the difference between the corresponding exact solution (*ψ*, *ω*) and the approximated solution of $\left(\tilde{\psi },\tilde{\omega }\right)$ (5.24)–(5.26), (A 4), is *O*(*θ*^3^) as well. See the electronic supplementary material for related error perturbation simulations.

To obtain the values of *Du*^(0)^(0) and *Du*^(1)^(0) in (A 4), we expand the underlying variables *ψ*′(*z*), *u*′(*z*), *ω* in orders of *θ* and solve the corresponding equations in succession. The system of exact equations (5.24)–(5.26), (A 1), (5.5), (5.8), (5.9) to zeroth order is

A 5 $$\begin{array}{rl} & \gamma \omega^{\left(0\right)}(D^{2}-k^{2})\psi^{\left(0\right)}=\frac{1}{Re}{\left(D^{2}-k^{2}\right)}^{2}\psi^{\left(0\right)}\;\text{on}\;0<z<2, \end{array}$$

A 6 $$\begin{array}{rl} & \omega^{\left(0\right)}r^{\prime }=-ik\kappa \bar{F}D^{2}\psi^{\left(0\right)}\;\text{at}\;z=0, \end{array}$$

A 7 $$\begin{array}{rl} & \psi^{\left(0\right)}=0,\;D\psi^{\left(0\right)}=0,\;u^{\left(0\right)}=0\;\text{at}\;z=2, \end{array}$$

A 8 $$\begin{array}{rl} & \psi^{\left(0\right)}=0,\;D\psi^{\left(0\right)}=0,\;u^{\left(0\right)}=-Lr^{\prime }D\bar{u}\;\text{at}\;z=0 \end{array}$$

A 9 $$\begin{array}{rl} \mathrm{and}\;\; & \gamma \omega^{\left(0\right)}u^{\left(0\right)}=ik\psi^{\left(0\right)}D\bar{u}+\frac{1}{Re}\left[D^{2}-k^{2}\right]u^{\left(0\right)}\;\text{on}\;0<z<2. \end{array}$$

We recall that *r*′ is a complex number that represents the amplitude and phase of the bed-form perturbation. Given the linearity of the system, *ψ*^(0)^(*z*) and *u*^(0)^ scale with *r*′.

We first solve for *ψ*^(0)^(*z*) and *ω*^(0)^. As discussed in §6, the system has multiple solutions, most of them corresponding to hydrodynamic modes. Substituting *ω*^(0)^ with a suitable negative value within the range indicated by figure 12 will lead to the computation of the hydrodynamic modes to zeroth order, although that system of equations is not algebraically solvable.

The sediment transport mode stands out from the other modes in sign, magnitude and the profile of the corresponding streamfunction *ψ*(*z*) (figure 5). Figure 12 suggests that as *θ* approaches 0, the sediment transport eigenvalue converges to 0. The choice of *ω*^(0)^ = 0 thus leads to the corresponding sediment transport mode of the system (A 5)–(A 8), namely *ψ*^(0)^(*z*) = 0. The equation for *u*^(0)^(*z*) then reduces to a second-order linear ODE with constant coefficients. The zeroth-order solution is given by

A 10 $$\omega^{\left(0\right)}=0,\;\psi^{\left(0\right)}(z)=0,\;u^{\left(0\right)}=-2Lr^{\prime }\frac{\exp(-kz)-\exp(k(z-4))}{1-\exp(-4k)},$$

where we substitute the steady-state term $D\bar{u}(0)=2$ in (A 9).

We compute the first correction term *Du*^(0)^(0) from (A 10),

A 11 $$Du^{\left(0\right)}(0)=\frac{2r^{\prime }Lk(1+\exp(-4k))}{1-\exp(-4k)}.$$

The value of *Du*^(0)^(0) is real and positive for all *k*, and its asymptotic properties are

A 12 $$\lim_{k\to 0}Du^{\left(0\right)}(0)=Lr^{\prime }\;\mathrm{and}\;\lim_{k\to \infty }\frac{Du^{\left(0\right)}(0)}{k}=2Lr^{\prime }.$$

The system of equations (5.24)–(5.26), (A 1), (5.5), (5.8), (5.9) to first order in *θ* is given by

A 13 $$\begin{array}{rl} 0 & ={\left(D^{2}-k^{2}\right)}^{2}\psi^{\left(1\right)}\;\text{on}\;0<z<2, \end{array}$$

A 14 $$\begin{array}{rl} \omega^{\left(1\right)}r^{\prime } & =-ik\kappa \bar{F}D^{2}\psi^{\left(1\right)}-ik\kappa V\bar{F}r^{\prime }Du^{\left(0\right)}\;\text{at}\;z=0, \end{array}$$

A 15 $$\begin{array}{rl} \psi^{\left(1\right)} & =0,\;D\psi^{\left(1\right)}=0,\;u^{\left(1\right)}=0\;\text{at}\;z=2, \end{array}$$

A 16 $$\begin{array}{rl} \psi^{\left(1\right)} & =0,\;D\psi^{\left(1\right)}=-2Lr^{\prime },\;u^{\left(1\right)}=0\;\text{at}\;z=0 \end{array}$$

A 17 $$\begin{array}{rl} \mathrm{and}\;\;\gamma \omega^{\left(1\right)}u^{\left(0\right)} & =-ik\bar{u}u^{\left(0\right)}+ik\psi^{\left(1\right)}D\bar{u}-\frac{2ik}{\prod Re}p^{\left(0\right)}+\frac{1}{Re}\left[D^{2}-k^{2}\right]u^{\left(1\right)}\;\text{on}\;0<z<2. \end{array}$$

Note that the zeroth-order pressure field *p*^(0)^(*z*) is uniformly 0 for the sediment transport mode since the underlying flow field is uniformly zero (*ψ*^(0)^(*z*) = 0).

The equations describe a linear ODE with constant coefficients for *ψ*^(1)^. Upon solving for *ψ*^(1)^, (A 14) provides us with the value of *ω*^(1)^. We note that *ω*^(1)^ is purely imaginary (see the electronic supplementary material). We then substitute *ψ*^(1)^(*z*) and *ω*^(1)^ in (A 17) and solve a linear ODE with constant coefficients to compute *u*^(1)^(*z*).

Our computations for the sediment transport eigenvalue show that *ω*^(0)^ = 0, and *ω*^(1)^_r_ = 0, where *ω* = *ω*_r_ + i*ω*_i_. Our expansion thus indicates that *ω*_r_ = *O*(*θ*^2^), which is consistent with figure 12. The above order analysis suggests that the zeroth-order system represents the flow field outside of the Squire plane. Given that *ω*^(1)^ is imaginary, the first-order system represents the bed-form transport due to diffusive processes in the flow field. It is the second-order system that represents the destabilizing advective processes within the film flow.

The full expression for the second correction term *Du*^(1)^(0) in (A 4) is given in the electronic supplementary material. This term is imaginary for all *k*, and its asymptotic properties are

A 18 $$\lim_{k\to 0}\frac{Du^{\left(1\right)}(0)}{k}=\frac{8}{15}ir^{\prime }LRe\;\mathrm{and}\;\lim_{k\to \infty }k^{2}Du^{\left(1\right)}(0)=\frac{1}{2}LRer^{\prime }.$$

Substituting the asymptotic properties (A 12) and (A 18) in (5.21) yields

A 19 $$\lim_{k\to 0}\frac{C(k,\theta )}{k}=-i\theta \kappa V\bar{F}Lr^{\prime }\;\mathrm{and}\;\lim_{k\to \infty }\frac{C(k,\theta )}{k^{2}}=-2i\theta \kappa V\bar{F}Lr^{\prime }.$$
