# Supplementary material for: Spatial heterogeneity in subglacial drainage driven by till erosion
Source: Proc Math Phys Eng Sci. 2019 Aug 14;475(2228):20190259. doi: 10.1098/rspa.2019.0259 (PMC6735472; doi:10.1098/rspa.2019.0259)
Supplement: Mathematical Derivations [file rspa20190259supp1.pdf]

Electronic Supplementary Material: Spatial Heterogeneity in Subglacial Drainage Driven by Till Erosion.

Indraneel Kasmalkar, Elisa Mantelli, Jenny Suckale

Main article:
Kasmalkar I, Mantelli E, Suckale J. 2019 Spatial heterogeneity in subglacial drainage driven by till erosion. Proc. R. Soc. A 20190259. http://dx.doi.org/10.1098/rspa.2019.0259. Accepted on July 15, 2019.

S1 Linearization of the Exner Equation

We introduce the perturbation $\exp(ik_1x + ik_2y + \omega t)$ into the non-dimensional Exner equation (3.3) from the manuscript. Omitting the star notation for non-dimensional variables,

$$\omega r' = -ik_1 q_1' - ik_2 q_2', \quad \mathbf{q}' = \overline{F}\hat{\boldsymbol{\tau}}' + \mathrm{S}|\boldsymbol{\tau}|'\mathrm{d}\overline{F}\hat{\overline{\boldsymbol{\tau}}}. \tag{S.1}$$

Here, $\overline{F}$ and $\mathrm{d}\overline{F}$ are steady–state sediment transport values derived in for $\overline{\boldsymbol{\tau}} = (1, 0)$,

$$\overline{F} = F(\mathrm{S}|\overline{\boldsymbol{\tau}}|) = A(\mathrm{S} - 0.12)^m, \quad \mathrm{d}\overline{F} = \frac{dF}{d|\boldsymbol{\tau}|}(\mathrm{S}|\overline{\boldsymbol{\tau}}|) = Am(\mathrm{S} - 0.12)^{m-1}, \quad A = 8, m = 1.5. \tag{S.2}$$

The non-dimensional bed stress $\boldsymbol{\tau} = (\tau_1, \tau_2)$ and unit bed stress vectors are given by (2.8),

$$\tau_i = \mathbf{t}_i^T \left(\nabla\mathbf{u} + \nabla\mathbf{u}^T\right)\mathbf{n}, \quad \hat{\boldsymbol{\tau}} = \frac{\boldsymbol{\tau}}{|\boldsymbol{\tau}|}, \quad \text{at} \quad z = r. \tag{S.3}$$

The vectors $\mathbf{t}_1, \mathbf{t}_2$ are the unit tangent vectors to the bed in the x- and y- directions respectively, and $\mathbf{n}$ is the normal surface vector for the bed $z = r(x, y, t)$,

$$\mathbf{t}_1 = \frac{\left(1, 0, \frac{\partial r}{\partial x}\right)}{\sqrt{1 + \left(\frac{\partial r}{\partial x}\right)^2}}, \quad \mathbf{t}_2 = \frac{\left(1, 0, \frac{\partial r}{\partial y}\right)}{\sqrt{1 + \left(\frac{\partial r}{\partial y}\right)^2}}, \quad \mathbf{n} = \frac{\left(-\frac{\partial r}{\partial x}, -\frac{\partial r}{\partial y}, 1\right)}{\sqrt{1 + \left(\frac{\partial r}{\partial x}\right)^2 + \left(\frac{\partial r}{\partial y}\right)^2}}. \tag{S.4}$$

After introducing the perturbation given in (5.1-5.3), these vectors take the form,

$$\mathbf{t}_1 = (1, 0, 0) + \varepsilon ik_1 r'(0, 0, 1), \quad \mathbf{t}_2 = (1, 0, 0) + \varepsilon ik_2 r'(0, 0, 1), \quad \mathbf{n} = (0, 0, 1) + \varepsilon(-ik_1 r', -ik_2 r', 0). \tag{S.5}$$

We evaluate the non-dimensional stress terms at both the steady state and their perturbations,

$$\overline{\boldsymbol{\tau}} = (1, 0), \quad \boldsymbol{\tau}' = (\mathrm{D}u' + ik_1 w', \mathrm{D}v' + ik_2 w'), \quad \hat{\overline{\boldsymbol{\tau}}} = (1, 0), \quad \hat{\boldsymbol{\tau}}' = (0, \mathrm{D}v' + ik_2 w'). \tag{S.6}$$

Care needs to be taken for the computation for $\hat{\boldsymbol{\tau}}' = \left(\frac{\boldsymbol{\tau}}{|\boldsymbol{\tau}|}\right)'$. The perturbation of $|\boldsymbol{\tau}|$ is given by $|\boldsymbol{\tau}|' = 1 + \varepsilon\ \mathrm{Real}\,(\mathrm{D}u' + ik_1 w')$.

Thus,

$$\mathbf{q}' = \left([\mathrm{D}u' + ik_1 w']\,\mathrm{S}\mathrm{d}\overline{F}\ \ ,\ \ \overline{F}\,[\mathrm{D}v' + ik_2 w']\right), \tag{S.7}$$

which yields,

$$\omega r' = -ik_1 \mathrm{S}\kappa\mathrm{d}\overline{F}\,[\mathrm{D}u' + ik_1 w'] - ik_2\kappa\overline{F}\,[\mathrm{D}v' + ik_2 w']\,. \tag{S.8}$$

We use the boundary conditions at $z = 0$ to remove the vertical velocity terms, $w' = 0$, to obtain the linearized Exner equation (5.10),

$$\omega r' = -ik_1 \mathrm{S}\kappa\mathrm{d}\overline{F}\mathrm{D}u' - ik_2\kappa\overline{F}\mathrm{D}v'. \tag{S.9}$$

S2 Small L Approximation

In equation (3.9), we invoke the assumption $\mathrm{L} \ll 1$ to simplify the boundary conditions at the till-water interface. We provide the mathematical details for performing this simplification.

The exact boundary condition for the vertical velocity w at the till-water interface, when linearized, projected onto the Squire plane and expressed in terms of the streamfunction ψ, is

$$\psi = \mathrm{L}\gamma\omega r' \quad \text{at} \quad z = 0. \tag{S.10}$$

We write $\mathrm{L} = \Lambda^2$. This is motivated by (3.7), which suggests that $\kappa = O\left(\frac{1}{\sqrt{L}}\right)$. We impose $\mathrm{S} = O(1)$ and express the other dependent non-dimensional parameters from (3.7) in order of Λ,

$$\gamma = O(\Lambda^2), \quad \kappa = O(\Lambda^{-1}), \quad \Pi = O(\Lambda^{-1}). \tag{S.11}$$

The scaling for Π results from our assumption $\mathrm{S} = O(1)$.

Similar to the analysis for θ in the Appendix, we perform a cubic expansion in Λ,

$$\omega = \omega^{(0)} + \Lambda\omega^{(1)} + \Lambda^2\omega^{(2)} + \Lambda^3\omega^{(3)} + O(\Lambda^4), \tag{S.12}$$

$$f(z) = f^{(0)}(z) + \Lambda f^{(1)}(z) + \Lambda^2 f^{(2)}(z) + \Lambda^3 f^{(3)}(z) + O(\Lambda^4), \tag{S.13}$$

where $f(z)$ stands for a generic variable $u', v', w', p', \mathcal{U}', \psi$. The equations for the system (5.25 - 5.28), are given by

$$0 = \frac{1}{\mathrm{Re}}(\mathrm{D}^2 - k^2)^2\psi^{(0)} \quad \text{on} \quad 0 < z < 2, \tag{S.14}$$

$$0 = -ik\kappa\overline{F}\mathrm{D}^2\psi^{(0)} \quad \text{at} \quad z = 0, \tag{S.15}$$

$$\psi^{(0)} = 0, \quad \mathrm{D}\psi^{(0)} = 0 \quad \text{at} \quad z = 2, \tag{S.16}$$

$$\psi^{(0)} = 0, \quad \mathrm{D}\psi^{(0)} = 0 \quad \text{at} \quad z = 0. \tag{S.17}$$

In simplifying (S.15) we multiply by Λ, and thus retain the term with κ only.

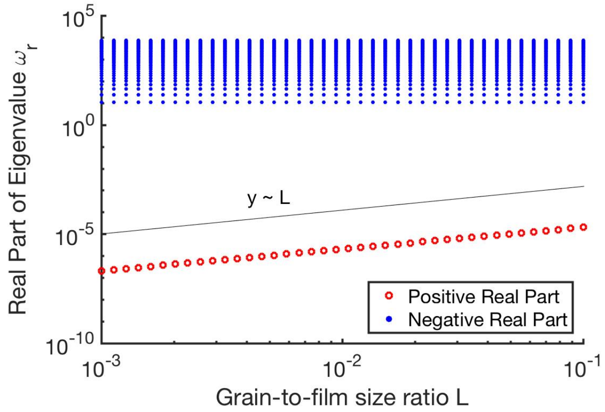

Figure S1: Numerical solution of sediment transport eigenvalue of the system of equations (5.25-5.28) varies as $O(\mathrm{L})$, while the hydrodynamic eigenvalues remain constant. $\mathrm{Re} = 20$, $\alpha = 0.001$, $k = 1$, $\theta = 0.01$.

In Figure S1, a study of how the numerical solution of the sediment transport eigenvalue $\tilde{\omega}$ varies with L shows that $\tilde{\omega} = O(\mathrm{L}) = O(\Lambda^2)$. Hence, to obtain the zero-th order solution to the system for the sediment transport eigenvalue, we choose $\omega^{(0)} = 0$. This yields the solution $\psi^{(0)} = 0$ to the above system.

The first-order system in Λ reads,

$$0 = \frac{1}{\mathrm{Re}}(\mathrm{D}^2 - k^2)^2\psi^{(1)} \quad \text{on} \quad 0 < z < 2, \tag{S.18}$$

$$\omega^{(0)} = -ik\kappa\overline{F}\mathrm{D}^2\psi^{(1)} + C^{(0)}(k,\theta) \quad \text{at} \quad z = 0, \tag{S.19}$$

$$\psi^{(1)} = 0, \quad \mathrm{D}\psi^{(1)} = 0 \quad \text{at} \quad z = 2, \tag{S.20}$$

$$\psi^{(1)} = 0, \quad \mathrm{D}\psi^{(1)} = 0 \quad \text{at} \quad z = 0. \tag{S.21}$$

In (S.19), we set the perturbation constant $r' = 1$ and we again multiply by Λ, which retains the terms $\omega^{(0)}$ and $C^{(0)}(k,\theta)$. From the zero-th order analysis, we note that $\omega^{(0)} = 0$, and the Appendix suggests that $C^{(0)}(k,\theta) = 0$. The solution we yield again, is $\omega^{(1)} = 0$ and $\psi^{(1)} = 0$.

Consistent with Figure S1, we see that $\omega = O(\Lambda^2)$, and also $\psi = O(\Lambda^2)$.

Reducing the various terms in our manuscript to error $O(\Lambda^4)$ leads to the set of equations (5.25-5.28). We now compile a list of various terms in the manuscript and highlight their L–order.

$$\omega^\star = \mathrm{L}\gamma\frac{\partial r^*}{\partial t^*} = O(\Lambda^4) = 0, \qquad \text{equation (2.1)},$$

$$-\mathrm{L}\sin(\theta)\mathrm{D}\overline{u} = O(\Lambda^2), \qquad \text{equation (5.26)},$$

$$a_1 = \overline{F}\mathrm{L}\gamma\kappa\sin(\theta) = O(\Lambda^3), \qquad \text{equation (6.4)},$$

$$b_1 = \overline{F}\mathrm{L}\gamma\kappa\sin(\theta) = O(\Lambda^3), \qquad \text{equation (6.13)}.$$

We also remark that the computations for $C(k,\theta)$ in the Appendix as well as in Section S5 have error at most $O(\Lambda^4)$.

S3 Numerics

For reference, we write the main system of equations (5.24-5.27) from the manuscript,

$$\gamma\omega\left[\mathrm{D}^2 - k^2\right]\psi = -ik_1\left[\overline{u}\mathrm{D}^2\psi - \psi\mathrm{D}^2\overline{u} - k^2\overline{u}\psi\right] + \frac{1}{\mathrm{Re}}\left[\mathrm{D}^2 - k^2\right]^2\psi, \tag{S.7}$$

$$\mathrm{D}\psi = 0, \quad \psi = 0, \quad \text{at } z = 2, \tag{S.8}$$

$$\mathrm{D}\psi = -\sin(\theta)\mathrm{L}\mathrm{D}\overline{u}r', \quad \psi = 0, \quad \text{at } z = 0, \tag{S.9}$$

$$\omega r' = -ik\kappa\overline{F}\mathrm{D}^2\psi \quad \text{at} \quad z = 0, \tag{S.10}$$

where $\psi(z)$ is the streamfunction, z corresponds to the coordinate along the film depth, Re is the Reynolds number, $\overline{u}(z)$ is the steady state velocity along the x-direction, r' is the bed-form perturbation amplitude, k is the perturbation wavenumber, $\overline{F}$ is the steady state non-dimensional bed-load flux value, θ is the Squire angle, $k_1 = k\sin\theta$ and κ is a non-dimensional variable that connects the model scaling to that of the standard bedload transport scaling. We present the details of spectral Galerkin solver for the equations (5.24 - 5.27). For the purpose of the solver, we perform the translation $\zeta = z - 1$. We define the modified Sobolev space,

$$H^2_{\pm1}[-1,1] = \left\{\varphi \in L^2[-1,1] : \quad \varphi(\pm1) = 0, \frac{d\varphi}{d\zeta}(\pm1) = 0, \quad \frac{d^j\varphi}{d\zeta^j} \in L^2[-1,1], \quad 0 \le j \le 2\right\}, \tag{S.22}$$

where $L^2[-1,1]$ is the space of all square-integrable functions on $-1 \le \zeta \le 1$.

We write (5.24) in weak form by integrating against $\varphi \in H^2_1[-1,1]$,

$$\omega M(\psi,\varphi) = A(\psi,\varphi), \tag{S.23}$$

where $M(\psi,\phi)$ and $A(\psi,\phi)$ are the mass and the stiffness bilinear forms, respectively,

$$M = \gamma\left[I_{20} - k^2 I_{00}\right], \quad A = ik_1\left[U_{200} - U_{020} + k^2 U_{000}\right] + \frac{1}{\mathrm{Re}}\left[I_{22} - 2k^2 I_{20} + k^4 I_{00}\right], \tag{S.24}$$

$$\text{and,} \quad I_{j_1j_2}(\psi,\varphi)\int_{-1}^{1}\frac{d^{j_1}\psi}{d\zeta^{j_1}}\frac{d^{j_2}\varphi}{d\zeta^{j_2}}d\zeta, \quad U_{j_1j_2j_3}(\psi,\varphi) = \int_{-1}^{1}\frac{d^{j_1}\overline{u}}{d\zeta^{j_1}}\frac{d^{j_2}\psi}{d\zeta^{j_2}}\frac{d^{j_3}\varphi}{d\zeta^{j_3}}d\zeta. \tag{S.25}$$

In (S.24) we use integration by parts, combined with boundary terms equaling zero due to (S.22).

We approximate the solution space for ψ by the finite dimensional subspace,

$$V_N = \text{Span}\{\psi_j : -1 \leq j \leq N\}, \tag{S.26}$$

where, for $1 \leq j \leq N$, we define ψ_j as the double-integrated Legendre polynomial L_{j+1} such that $\psi_j(\pm 1) = \frac{d\psi_j}{d\zeta}(\pm 1) = 0$, namely,

$$\psi_j = \sqrt{j+\frac{3}{2}}\left(\frac{L_{j+3}-L_{j+1}}{(2j+3)(2j+5)} - \frac{L_{j+1}-L_{j-1}}{(2j+1)(2j+3)}\right), \tag{S.27}$$

and ψ_0, ψ_1 correspond to two low-degree polynomials, linearly independent from the other ψ_j, to incorporate the two boundary conditions (5.26) at the till-water interface,

$$\psi_0(z) = (\zeta-1)^2, \quad \psi_{-1}(z) = (\zeta-1)^2(\zeta+2). \tag{S.28}$$

We write the solution as $\psi = \sum_{j=-1}^{N} a_j \psi_j$. Note that the ice-water boundary conditions (5.25) would be automatically satisfied by any such ψ. We incorporate the boundary conditions (5.26) and the Exner equation (5.27) in strong form.

We approximate the test function space $H^2_{\pm 1}[-1,1]$ by the finite dimensional subspace W_N, which consists of just the standard basis functions without the low-degree polynomials.

$$W_N = \text{Span}\{\psi_j : 1 \leq j \leq N\}. \tag{S.29}$$

We reformulate the bilinear forms A, I, U and M in (S.23) as $(N+3)\times(N+3)$ matrices. The $N+3$ columns stand the unknowns represented by $\mathbf{x} = (a_{-1}, a_0, a_1, ..., a_N, r')$. The $N+3$ rows stand for integration against the N test functions of W_N, plus three additional rows that describe the two boundary conditions (5.26) and the Exner equation (5.27). With we obtain a finite dimensional eigenvalue problem, $A\mathbf{x} = \omega M\mathbf{x}$, which we solve using the Matlab *eig* routine.

S4 Asymptotic Analysis

S4.1 Short–Wavelength Advection

The analytical solution of the reduced model for the short wavelength advection-diffusion regime in Section 6(c) is given by,

$$\psi = \frac{2\int_0^z \int_v^\infty e^{2v-s-z}\text{Ai}(c^{-1}s+c^2)dsdv}{\int_0^\infty e^{-s}\text{Ai}(c^{-1}s+c^2)ds}, \quad \omega = \frac{-2i\text{Ai}(c^2)}{\int_0^\infty e^{-s}\text{Ai}\left(c^{-1}s+c^2\right)ds}, \tag{S.30}$$

where $c = \frac{1}{\sqrt[3]{2ia_2}}$ with $\arg(c) = -\frac{\pi}{6}$, and $\text{Ai}(z)$ is one of the two standard linearly independent solutions of the system $\text{D}^2 f = zf$. The integrals in (S.30) converge due to the exponential decay rate of $\text{Ai}(z)$ for $-\frac{\pi}{3} < z < \frac{\pi}{3}$.

We compare the theoretical value of ω, given above, and the rescaled numerical results, $\omega_{\text{num}} = \frac{\omega}{\overline{F}\text{L}k^2\sin\theta}$ in Figure S2.

S4.2 Short–Wavelength Acceleration

The analytical solution for the reduced model in Section 6(d) is given by,

$$\psi = \frac{2\left[e^{-z} - e^{-z\sqrt{1+a_1\omega}}\right]}{1-\sqrt{1+a_1\omega}}, \quad \omega = -2a_1 + 2i - 2i\text{V} + 2i\sqrt{(1+a_1 i)^2 - 2a_1 i\text{V}} \tag{S.31}$$

where $a_1 = \overline{F}\text{LRe}\gamma\kappa\sin\theta$. In the above equation, the value of ω can be derived algebraically by substituting the expression for ψ in (S.31) into the reduced Exner equation (6.10). That leads to a quadratic in ω which naturally has two solutions as a results of the term $\sqrt{(1+a_1 i)^2 - 2a_1 i\text{V}}$. Assuming that ω is continuous in its parameters, we choose the square-root that varies continuously with $a_1 > 0$ and $\text{V} \geq 0$. This square-root is characterized as the one with non-negative imaginary part.

We compare the theoretical value of ω, given above, and the rescaled numerical results, $\omega_{\text{num}} = \frac{\omega}{\overline{F}\text{L}k^2\sin\theta}$ in Figure S3. We choose $V = 1$.

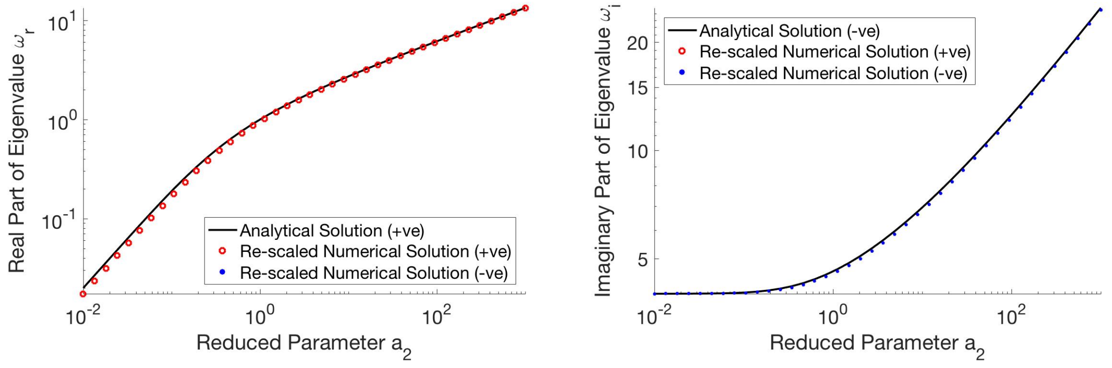

(a) Comparison for the real part, ω_r. (b) Comparison for the imaginary part, ω_i.

Figure S2: Analytical and re-scaled numerical solutions for the short wavelength advection regime.

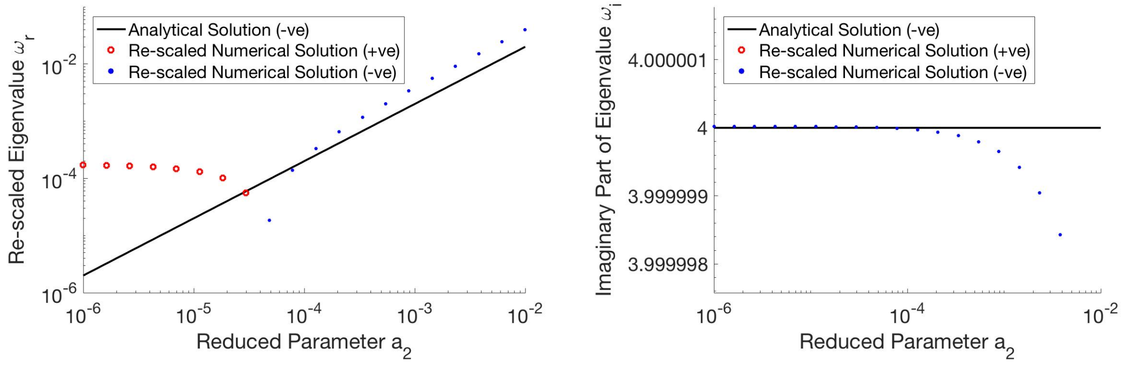

(a) Comparison for the real part, ω_r. (b) Comparison for the imaginary part, ω_i.

Figure S3: Analytical and re-scaled numerical solutions for the short wavelength acceleration regime.

S5 θ-Expansions of the System of Equations

As laid out in the Appendix, we perform a quadratic expansion of the system of equations (5.25-5.27,9.1, 5.5, 5.8, 5.9) to compute the necessary correction terms in the Exner equation (9.3). We solve the zero-th and first-order system of equations using the Matlab symbolic toolbox.

S5.1 Perturbation Study for the System of Equations

We rewrite the main system of equations for meltwater film stability (5.25-2.28),

$$\gamma\omega\left[\mathrm{D}^2-k^2\right]\psi=-ik\theta\left[\overline{u}\mathrm{D}^2\psi-\psi\mathrm{D}^2\overline{u}-k^2\overline{u}\psi\right]+\frac{1}{\mathrm{Re}}\left[\mathrm{D}^2-k^2\right]^2\psi,\quad\text{on}\quad 0<z<2. \tag{S.32}$$

$$\mathrm{D}\psi=0,\qquad \psi=0,\qquad \text{at } z=2, \tag{S.33}$$

$$\mathrm{D}\psi=-\theta\mathrm{L}\mathrm{D}\overline{u}r',\qquad \psi=0,\qquad \text{at } z=0, \tag{S.34}$$

$$\omega r'=-ik\kappa\overline{F}\mathrm{D}^2\psi+C(k,\theta)+\delta\quad\text{at}\quad z=0. \tag{S.35}$$

In (5.27) there is an additional constant δ. Figure S4 supports the statement in the Appendix that an error of δ in this system of equations leads to a corresponding error of $O(\delta)$ between the numerical solution $(\tilde{\psi},\tilde{\omega})$ and the exact solution (ψ,ω). The error is measured in the discrete L^1 norm as $E(\delta)=\|\psi-\psi_\delta\|+|\omega-\omega_\delta|$.

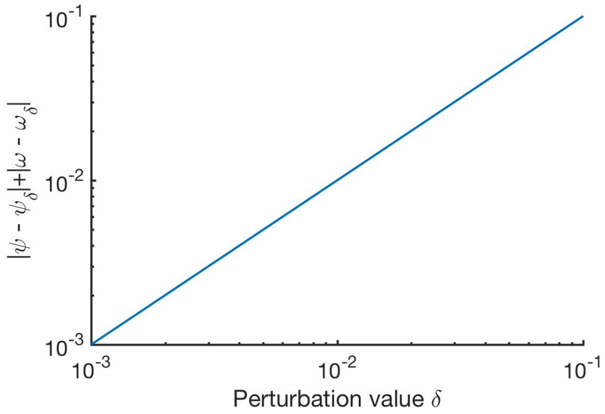

Figure S4: Perturbation study that shows that an error δ in the system of equations leads to an $O(\delta)$ error in the solution. $k = 1$, $\theta = 0.01$, $\mathrm{Re} = 20$, $\mathrm{L} = 0.001$, $\alpha = 0.001$.

S5.2 Symbolic expressions

The symbolic expressions for the solutions for the sediment transport mode are given below. RM stands for κ and RT stands for γ. Consistent with the approximation for L in Section S2, the numerical solver used in Section 6 only retains the terms that have error at most $O(\mathrm{L}^2)$.

```
omega^(0) = 0

psi^(0) = 0

u^(0) = (2*L*exp(2*k)*exp(-k*z))/(exp(-2*k) - exp(2*k)) - (2*L*exp(-2*k)*exp(k*z))/(
    exp(-2*k) - exp(2*k))

psi^(1) = -(2*L*(z*exp(4*k) - z*exp(8*k) - z*exp(2*k*z) + z*exp(2*k*(z + 2))) + 2*L*k
    *(8*exp(2*k*(z + 2)) - 8*exp(4*k) + 4*z*exp(4*k) - 4*z*exp(2*k*(z + 2))))/(2*exp(k
    *(z + 4)) - exp(k*(z + 8)) - exp(k*z) + 16*k^2*exp(k*(z + 4)))

omega^(1) = - F*RM*k*((2*L*(4*k - 4*k*exp(4*k)) - 2*L*k*(32*k^2*exp(4*k) - 16*k*exp(4*
    k)))/(2*exp(4*k) - exp(8*k) + 16*k^2*exp(4*k) - 1) + (2*(2*L*(exp(8*k) - 2*exp(4*k
    ) + 1) - 32*L*k^2*exp(4*k))*(k - 16*k^3*exp(4*k) - 2*k*exp(4*k) + k*exp(8*k)))/(2*
    exp(4*k) - exp(8*k) + 16*k^2*exp(4*k) - 1)^2)*1i - (F*L*RM*V*k^2*(exp(4*k) + 1)*2i
    )/(exp(4*k) - 1)

u^(1) = (L*Re*exp(k*z)*(exp(8*k)*9i - exp(4*k)*3i - exp(12*k)*9i + exp(16*k)*3i + k^2*
    exp(4*k)*72i - k^3*exp(4*k)*96i - k^2*exp(8*k)*192i + k^3*exp(8*k)*288i + k^2*exp
    (12*k)*120i - k^3*exp(12*k)*192i - k*exp(4*k)*24i + k*exp(8*k)*48i - k*exp(12*k)
    *24i - k^2*z^2*exp(4*k)*6i - k^3*z^2*exp(4*k)*48i + k^2*z^2*exp(8*k)*18i + k^3*z
    ^2*exp(8*k)*96i - k^4*z^2*exp(8*k)*96i - k^2*z^2*exp(12*k)*18i - k^3*z^2*exp(12*k)
    *48i + k^4*z^2*exp(12*k)*96i + k^2*z^2*exp(16*k)*6i - k*z*exp(4*k)*6i + k*z*exp(8*
    k)*18i - k*z*exp(12*k)*18i + k*z*exp(16*k)*6i - k^4*z*exp(4*k + 2*k*z)*192i + k^4*
    z*exp(8*k + 2*k*z)*384i - k^4*z*exp(12*k + 2*k*z)*192i - k^3*z^3*exp(2*k*z)*4i - k
    ^2*z*exp(4*k)*48i + k^3*z*exp(4*k)*144i + k^2*z*exp(8*k)*96i - k^3*z*exp(8*k)*384i
     + k^4*z*exp(8*k)*192i - k^2*z*exp(12*k)*48i + k^3*z*exp(12*k)*240i - k^4*z*exp
    (12*k)*192i + k^3*z^3*exp(4*k + 2*k*z)*12i + k^4*z^2*exp(4*k + 2*k*z)*144i - k^4*z
    ^3*exp(4*k + 2*k*z)*32i + k^5*z^2*exp(4*k + 2*k*z)*192i - k^5*z^3*exp(4*k + 2*k*z)
    *64i - k^3*z^3*exp(8*k + 2*k*z)*12i - k^4*z^2*exp(8*k + 2*k*z)*288i + k^4*z^3*exp
    (8*k + 2*k*z)*64i - k^5*z^2*exp(8*k + 2*k*z)*192i + k^5*z^3*exp(8*k + 2*k*z)*64i +
```

```
 k^3*z^3*exp(12*k + 2*k*z)*4i + k^4*z^2*exp(12*k + 2*k*z)*144i - k^4*z^3*exp(12*k
+ 2*k*z)*32i - F*L*RM*RT*k^3*exp(4*k)*24i + F*L*RM*RT*k^3*exp(8*k)*24i - F*L*RM*RT
*k^4*exp(8*k)*192i + F*L*RM*RT*k^3*exp(12*k)*24i + F*L*RM*RT*k^4*exp(12*k)*192i -
F*L*RM*RT*k^3*exp(16*k)*24i - F*L*RM*RT*k^4*z*exp(2*k*z)*48i + F*L*RM*RT*k^4*z*exp
(4*k + 2*k*z)*48i - F*L*RM*RT*k^5*z*exp(4*k + 2*k*z)*384i + F*L*RM*RT*k^4*z*exp(8*
k + 2*k*z)*48i + F*L*RM*RT*k^5*z*exp(8*k + 2*k*z)*384i - F*L*RM*RT*k^4*z*exp(12*k
+ 2*k*z)*48i - F*L*RM*RT*V*k^3*exp(4*k)*12i + F*L*RM*RT*V*k^3*exp(8*k)*12i + F*L*
RM*RT*V*k^5*exp(8*k)*192i + F*L*RM*RT*V*k^3*exp(12*k)*12i + F*L*RM*RT*V*k^5*exp
(12*k)*192i - F*L*RM*RT*V*k^3*exp(16*k)*12i + F*L*RM*RT*V*k^4*z*exp(4*k + 2*k*z)
*24i + F*L*RM*RT*V*k^6*z*exp(4*k + 2*k*z)*384i + F*L*RM*RT*V*k^4*z*exp(8*k + 2*k*z
)*24i + F*L*RM*RT*V*k^6*z*exp(8*k + 2*k*z)*384i - F*L*RM*RT*V*k^4*z*exp(12*k + 2*k
*z)*24i - F*L*RM*RT*V*k^4*z*exp(2*k*z)*24i))/(12*k^3*(exp(2*k*z) - 4*exp(4*k + 2*k
*z) + 6*exp(8*k + 2*k*z) - 4*exp(12*k + 2*k*z) + exp(16*k + 2*k*z) - 16*k^2*exp(4*
k + 2*k*z) + 32*k^2*exp(8*k + 2*k*z) - 16*k^2*exp(12*k + 2*k*z))) - (L*Re*exp(-k*z
)*(exp(2*k*z)*3i - exp(4*k + 2*k*z)*9i + exp(8*k + 2*k*z)*9i - exp(12*k + 2*k*z)*3
i + k*exp(4*k + 2*k*z)*24i - k*exp(8*k + 2*k*z)*48i + k*exp(12*k + 2*k*z)*24i + k
^2*exp(4*k + 2*k*z)*120i + k^3*exp(4*k + 2*k*z)*192i - k^2*exp(8*k + 2*k*z)*192i -
 k^3*exp(8*k + 2*k*z)*288i + k^2*exp(12*k + 2*k*z)*72i + k^3*exp(12*k + 2*k*z)*96i
 + k*z*exp(4*k + 2*k*z)*18i - k*z*exp(8*k + 2*k*z)*18i + k*z*exp(12*k + 2*k*z)*6i
- k^3*z^3*exp(4*k)*4i + k^4*z^2*exp(4*k)*144i - k^4*z^3*exp(4*k)*32i + k^3*z^3*exp
(8*k)*12i - k^4*z^2*exp(8*k)*288i + k^4*z^3*exp(8*k)*64i + k^5*z^2*exp(8*k)*192i -
 k^5*z^3*exp(8*k)*64i - k^3*z^3*exp(12*k)*12i + k^4*z^2*exp(12*k)*144i - k^4*z^3*
exp(12*k)*32i - k^5*z^2*exp(12*k)*192i + k^5*z^3*exp(12*k)*64i + k^3*z^3*exp(16*k)
*4i - k^2*z*exp(4*k + 2*k*z)*48i - k^3*z*exp(4*k + 2*k*z)*240i - k^4*z*exp(4*k +
2*k*z)*192i + k^2*z*exp(8*k + 2*k*z)*96i + k^3*z*exp(8*k + 2*k*z)*384i + k^4*z*exp
(8*k + 2*k*z)*192i - k^2*z*exp(12*k + 2*k*z)*48i - k^3*z*exp(12*k + 2*k*z)*144i +
k^2*z^2*exp(2*k*z)*6i - k^4*z*exp(4*k)*192i + k^4*z*exp(8*k)*384i - k^4*z*exp(12*k
)*192i - k^2*z^2*exp(4*k + 2*k*z)*18i + k^3*z^2*exp(4*k + 2*k*z)*48i + k^4*z^2*exp
(4*k + 2*k*z)*96i + k^2*z^2*exp(8*k + 2*k*z)*18i - k^3*z^2*exp(8*k + 2*k*z)*96i -
k^4*z^2*exp(8*k + 2*k*z)*96i - k^2*z^2*exp(12*k + 2*k*z)*6i + k^3*z^2*exp(12*k +
2*k*z)*48i - k*z*exp(2*k*z)*6i + F*L*RM*RT*k^3*exp(2*k*z)*24i - F*L*RM*RT*k^3*exp
(4*k + 2*k*z)*24i + F*L*RM*RT*k^4*exp(4*k + 2*k*z)*192i - F*L*RM*RT*k^3*exp(8*k +
2*k*z)*24i - F*L*RM*RT*k^4*exp(8*k + 2*k*z)*192i + F*L*RM*RT*k^3*exp(12*k + 2*k*z)
*24i - F*L*RM*RT*k^4*z*exp(4*k)*48i + F*L*RM*RT*k^4*z*exp(8*k)*48i - F*L*RM*RT*k
^5*z*exp(8*k)*384i + F*L*RM*RT*k^4*z*exp(12*k)*48i + F*L*RM*RT*k^5*z*exp(12*k)*384
i - F*L*RM*RT*k^4*z*exp(16*k)*48i + F*L*RM*RT*V*k^3*exp(2*k*z)*12i - F*L*RM*RT*V*k
^3*exp(4*k + 2*k*z)*12i - F*L*RM*RT*V*k^5*exp(4*k + 2*k*z)*192i - F*L*RM*RT*V*k^3*
exp(8*k + 2*k*z)*12i - F*L*RM*RT*V*k^5*exp(8*k + 2*k*z)*192i + F*L*RM*RT*V*k^3*exp
(12*k + 2*k*z)*12i - F*L*RM*RT*V*k^4*z*exp(4*k)*24i + F*L*RM*RT*V*k^4*z*exp(8*k)
*24i + F*L*RM*RT*V*k^6*z*exp(8*k)*384i + F*L*RM*RT*V*k^4*z*exp(12*k)*24i + F*L*RM*
RT*V*k^6*z*exp(12*k)*384i - F*L*RM*RT*V*k^4*z*exp(16*k)*24i))/(12*k^3*(exp(4*k) -
1)^2*(2*exp(4*k) - exp(8*k) + 16*k^2*exp(4*k) - 1)) + (L*Re*exp(k*z)*(exp(6*k)*36i
 - exp(2*k)*3i - exp(10*k)*198i + exp(14*k)*660i - exp(18*k)*1485i + exp(22*k)
*2376i - exp(26*k)*2772i + exp(30*k)*2376i - exp(34*k)*1485i + exp(38*k)*660i -
exp(42*k)*198i + exp(46*k)*36i - exp(50*k)*3i - k^2*exp(6*k)*120i - k^3*exp(6*k)
*160i - k^4*exp(6*k)*64i + k^2*exp(10*k)*1176i + k^3*exp(10*k)*2816i + k^4*exp(10*
k)*6720i + k^5*exp(10*k)*5632i - k^2*exp(14*k)*5160i + k^6*exp(10*k)*2048i - k^3*
exp(14*k)*18208i - k^4*exp(14*k)*50624i - k^5*exp(14*k)*47616i + k^2*exp(18*k)
*13320i - k^6*exp(14*k)*69632i + k^3*exp(18*k)*63552i - k^7*exp(14*k)*57344i + k
^4*exp(18*k)*170688i - k^8*exp(14*k)*16384i + k^5*exp(18*k)*178176i - k^2*exp(22*k
)*22320i + k^6*exp(18*k)*366592i - k^3*exp(22*k)*138048i + k^7*exp(18*k)*278528i -
 k^4*exp(22*k)*328320i + k^8*exp(18*k)*81920i - k^5*exp(22*k)*387072i + k^9*exp
(18*k)*131072i + k^2*exp(26*k)*25200i - k^6*exp(22*k)*849920i + k^3*exp(26*k)
*198912i - k^7*exp(22*k)*565248i + k^4*exp(26*k)*389760i - k^8*exp(22*k)*147456i +
```

```
k^5*exp(26*k)*537600i - k^9*exp(22*k)*655360i - k^2*exp(30*k)*19152i + k^6*exp
(26*k)*1042432i - k^3*exp(30*k)*194880i + k^7*exp(26*k)*655360i - k^4*exp(30*k)
*287616i + k^8*exp(26*k)*81920i - k^5*exp(30*k)*494592i + k^9*exp(26*k)*1310720i +
k^2*exp(34*k)*9360i - k^6*exp(30*k)*677888i + k^3*exp(34*k)*129408i - k^7*exp(30*
k)*532480i + k^4*exp(34*k)*123264i + k^8*exp(30*k)*81920i + k^5*exp(34*k)*301056i
- k^9*exp(30*k)*1310720i - k^2*exp(38*k)*2520i + k^6*exp(34*k)*182272i - k^3*exp
(38*k)*56352i + k^7*exp(34*k)*344064i - k^4*exp(38*k)*22848i - k^8*exp(34*k)
*147456i - k^5*exp(38*k)*116736i + k^9*exp(34*k)*655360i + k^2*exp(42*k)*120i + k
^6*exp(38*k)*22528i + k^3*exp(42*k)*14848i - k^7*exp(38*k)*155648i - k^4*exp(42*k)
*2240i + k^8*exp(38*k)*81920i + k^5*exp(42*k)*26112i - k^9*exp(38*k)*131072i + k
^2*exp(46*k)*120i - k^6*exp(42*k)*20480i - k^3*exp(46*k)*1952i + k^7*exp(42*k)
*32768i + k^4*exp(46*k)*1344i - k^8*exp(42*k)*16384i - k^5*exp(46*k)*2560i - k^2*
exp(50*k)*24i + k^6*exp(46*k)*2048i + k^3*exp(50*k)*64i - k^4*exp(50*k)*64i - k*
exp(6*k)*48i + k*exp(10*k)*528i - k*exp(14*k)*2640i + k*exp(18*k)*7920i - k*exp
(22*k)*15840i + k*exp(26*k)*22176i - k*exp(30*k)*22176i + k*exp(34*k)*15840i - k*
exp(38*k)*7920i + k*exp(42*k)*2640i - k*exp(46*k)*528i + k*exp(50*k)*48i - F*L*RM*
RT*k^3*exp(2*k)*24i + F*L*RM*RT*k^3*exp(6*k)*240i - F*L*RM*RT*k^4*exp(6*k)*384i +
F*L*RM*RT*k^5*exp(6*k)*768i - F*L*RM*RT*k^3*exp(10*k)*1056i + F*L*RM*RT*k^4*exp
(10*k)*3648i - F*L*RM*RT*k^5*exp(10*k)*7680i + F*L*RM*RT*k^6*exp(10*k)*12288i + F*
L*RM*RT*k^3*exp(14*k)*2640i - F*L*RM*RT*k^7*exp(10*k)*6144i - F*L*RM*RT*k^4*exp
(14*k)*15360i + F*L*RM*RT*k^5*exp(14*k)*34560i - F*L*RM*RT*k^6*exp(14*k)*92160i -
F*L*RM*RT*k^3*exp(18*k)*3960i + F*L*RM*RT*k^7*exp(14*k)*86016i + F*L*RM*RT*k^4*exp
(18*k)*37440i - F*L*RM*RT*k^8*exp(14*k)*98304i - F*L*RM*RT*k^5*exp(18*k)*92160i +
F*L*RM*RT*k^6*exp(18*k)*294912i + F*L*RM*RT*k^3*exp(22*k)*3168i - F*L*RM*RT*k^7*
exp(18*k)*430080i - F*L*RM*RT*k^4*exp(22*k)*57600i + F*L*RM*RT*k^8*exp(18*k)
*540672i + F*L*RM*RT*k^5*exp(22*k)*161280i - F*L*RM*RT*k^9*exp(18*k)*393216i - F*L
*RM*RT*k^6*exp(22*k)*516096i + F*L*RM*RT*k^7*exp(22*k)*1118208i + F*L*RM*RT*k^4*
exp(26*k)*56448i - F*L*RM*RT*k^8*exp(22*k)*1179648i - F*L*RM*RT*k^5*exp(26*k)
*193536i + F*L*RM*RT*k^9*exp(22*k)*1966080i + F*L*RM*RT*k^6*exp(26*k)*516096i - F*
L*RM*RT*k^3*exp(30*k)*3168i - F*L*RM*RT*k^7*exp(26*k)*1720320i - F*L*RM*RT*k^4*exp
(30*k)*32256i + F*L*RM*RT*k^8*exp(26*k)*1228800i + F*L*RM*RT*k^5*exp(30*k)*161280i
- F*L*RM*RT*k^9*exp(26*k)*3932160i - F*L*RM*RT*k^6*exp(30*k)*258048i + F*L*RM*RT*
k^3*exp(34*k)*3960i + F*L*RM*RT*k^7*exp(30*k)*1634304i + F*L*RM*RT*k^4*exp(34*k)
*5760i - F*L*RM*RT*k^8*exp(30*k)*491520i - F*L*RM*RT*k^5*exp(34*k)*92160i + F*L*RM
*RT*k^9*exp(30*k)*3932160i - F*L*RM*RT*k^3*exp(38*k)*2640i - F*L*RM*RT*k^7*exp(34*
k)*946176i + F*L*RM*RT*k^4*exp(38*k)*5760i - F*L*RM*RT*k^8*exp(34*k)*147456i + F*L
*RM*RT*k^5*exp(38*k)*34560i - F*L*RM*RT*k^9*exp(34*k)*1966080i + F*L*RM*RT*k^6*exp
(38*k)*73728i + F*L*RM*RT*k^3*exp(42*k)*1056i + F*L*RM*RT*k^7*exp(38*k)*307200i -
F*L*RM*RT*k^4*exp(42*k)*4800i + F*L*RM*RT*k^8*exp(38*k)*196608i - F*L*RM*RT*k^5*
exp(42*k)*7680i + F*L*RM*RT*k^9*exp(38*k)*393216i - F*L*RM*RT*k^6*exp(42*k)*36864i
- F*L*RM*RT*k^3*exp(46*k)*240i - F*L*RM*RT*k^7*exp(42*k)*43008i + F*L*RM*RT*k^4*
exp(46*k)*1536i - F*L*RM*RT*k^8*exp(42*k)*49152i + F*L*RM*RT*k^5*exp(46*k)*768i +
F*L*RM*RT*k^6*exp(46*k)*6144i + F*L*RM*RT*k^3*exp(50*k)*24i - F*L*RM*RT*k^4*exp
(50*k)*192i - F*L*RM*RT*V*k^3*exp(2*k)*12i + F*L*RM*RT*V*k^3*exp(6*k)*120i - F*L*
RM*RT*V*k^4*exp(6*k)*96i + F*L*RM*RT*V*k^5*exp(6*k)*576i - F*L*RM*RT*V*k^3*exp(10*
k)*528i + F*L*RM*RT*V*k^4*exp(10*k)*864i - F*L*RM*RT*V*k^5*exp(10*k)*4608i + F*L*
RM*RT*V*k^6*exp(10*k)*4608i + F*L*RM*RT*V*k^3*exp(14*k)*1320i - F*L*RM*RT*V*k^7*
exp(10*k)*9216i - F*L*RM*RT*V*k^4*exp(14*k)*3360i + F*L*RM*RT*V*k^5*exp(14*k)
*15552i - F*L*RM*RT*V*k^6*exp(14*k)*32256i - F*L*RM*RT*V*k^3*exp(18*k)*1980i + F*L
*RM*RT*V*k^7*exp(14*k)*55296i + F*L*RM*RT*V*k^4*exp(18*k)*7200i - F*L*RM*RT*V*k^8*
exp(14*k)*73728i - F*L*RM*RT*V*k^5*exp(18*k)*27648i + F*L*RM*RT*V*k^9*exp(14*k)
*49152i + F*L*RM*RT*V*k^6*exp(18*k)*92160i + F*L*RM*RT*V*k^3*exp(22*k)*1584i - F*L
*RM*RT*V*k^7*exp(18*k)*129024i - F*L*RM*RT*V*k^4*exp(22*k)*8640i + F*L*RM*RT*V*k
^8*exp(18*k)*368640i + F*L*RM*RT*V*k^5*exp(22*k)*24192i - F*L*RM*RT*V*k^9*exp(18*k
```

```
)*196608i - F*L*RM*RT*V*k^6*exp(22*k)*129024i + F*L*RM*RT*V*k^10*exp(18*k)*393216i
 + F*L*RM*RT*V*k^7*exp(22*k)*129024i + F*L*RM*RT*V*k^4*exp(26*k)*4032i - F*L*RM*RT
*V*k^8*exp(22*k)*663552i + F*L*RM*RT*V*k^9*exp(22*k)*245760i + F*L*RM*RT*V*k^6*exp
(26*k)*64512i - F*L*RM*RT*V*k^10*exp(22*k)*1179648i - F*L*RM*RT*V*k^3*exp(30*k)
*1584i + F*L*RM*RT*V*k^4*exp(30*k)*4032i + F*L*RM*RT*V*k^8*exp(26*k)*368640i - F*L
*RM*RT*V*k^5*exp(30*k)*24192i + F*L*RM*RT*V*k^6*exp(30*k)*64512i + F*L*RM*RT*V*k
^10*exp(26*k)*786432i + F*L*RM*RT*V*k^3*exp(34*k)*1980i - F*L*RM*RT*V*k^7*exp(30*k
)*129024i - F*L*RM*RT*V*k^4*exp(34*k)*8640i + F*L*RM*RT*V*k^8*exp(30*k)*368640i +
F*L*RM*RT*V*k^5*exp(34*k)*27648i - F*L*RM*RT*V*k^9*exp(30*k)*245760i - F*L*RM*RT*V
*k^6*exp(34*k)*129024i + F*L*RM*RT*V*k^10*exp(30*k)*786432i - F*L*RM*RT*V*k^3*exp
(38*k)*1320i + F*L*RM*RT*V*k^7*exp(34*k)*129024i + F*L*RM*RT*V*k^4*exp(38*k)*7200i
 - F*L*RM*RT*V*k^8*exp(34*k)*663552i - F*L*RM*RT*V*k^5*exp(38*k)*15552i + F*L*RM*
RT*V*k^9*exp(34*k)*196608i + F*L*RM*RT*V*k^6*exp(38*k)*92160i - F*L*RM*RT*V*k^10*
exp(34*k)*1179648i + F*L*RM*RT*V*k^3*exp(42*k)*528i - F*L*RM*RT*V*k^7*exp(38*k)
*55296i - F*L*RM*RT*V*k^4*exp(42*k)*3360i + F*L*RM*RT*V*k^8*exp(38*k)*368640i + F*
L*RM*RT*V*k^5*exp(42*k)*4608i - F*L*RM*RT*V*k^9*exp(38*k)*49152i - F*L*RM*RT*V*k
^6*exp(42*k)*32256i + F*L*RM*RT*V*k^10*exp(38*k)*393216i - F*L*RM*RT*V*k^3*exp(46*
k)*120i + F*L*RM*RT*V*k^7*exp(42*k)*9216i + F*L*RM*RT*V*k^4*exp(46*k)*864i - F*L*
RM*RT*V*k^8*exp(42*k)*73728i - F*L*RM*RT*V*k^5*exp(46*k)*576i + F*L*RM*RT*V*k^6*
exp(46*k)*4608i + F*L*RM*RT*V*k^3*exp(50*k)*12i - F*L*RM*RT*V*k^4*exp(50*k)*96i))
/(12*k^3*(exp(-2*k) - exp(2*k))*(exp(4*k) - 1)^2*(2*exp(4*k) - exp(8*k) + 16*k^2*
exp(4*k) - 1)*(exp(4*k) - 4*exp(8*k) + 6*exp(12*k) - 4*exp(16*k) + exp(20*k) - 16*
k^2*exp(8*k) + 32*k^2*exp(12*k) - 16*k^2*exp(16*k))*(4*exp(4*k) - 6*exp(8*k) + 4*
exp(12*k) - exp(16*k) + 16*k^2*exp(4*k) - 32*k^2*exp(8*k) + 16*k^2*exp(12*k) - 1))
 - (L*Re*exp(-k*z)*(exp(10*k)*36i - exp(6*k)*3i - exp(14*k)*198i + exp(18*k)*660i
- exp(22*k)*1485i + exp(26*k)*2376i - exp(30*k)*2772i + exp(34*k)*2376i - exp(38*k
)*1485i + exp(42*k)*660i - exp(46*k)*198i + exp(50*k)*36i - exp(54*k)*3i - k^2*exp
(6*k)*24i - k^3*exp(6*k)*64i - k^4*exp(6*k)*64i + k^2*exp(10*k)*120i + k^3*exp(10*
k)*1952i + k^4*exp(10*k)*1344i + k^5*exp(10*k)*2560i + k^2*exp(14*k)*120i + k^6*
exp(10*k)*2048i - k^3*exp(14*k)*14848i - k^4*exp(14*k)*2240i - k^5*exp(14*k)*26112
i - k^2*exp(18*k)*2520i - k^6*exp(14*k)*20480i + k^3*exp(18*k)*56352i - k^7*exp
(14*k)*32768i - k^4*exp(18*k)*22848i - k^8*exp(14*k)*16384i + k^5*exp(18*k)*116736
i + k^2*exp(22*k)*9360i + k^6*exp(18*k)*22528i - k^3*exp(22*k)*129408i + k^7*exp
(18*k)*155648i + k^4*exp(22*k)*123264i + k^8*exp(18*k)*81920i - k^5*exp(22*k)
*301056i + k^9*exp(18*k)*131072i - k^2*exp(26*k)*19152i + k^6*exp(22*k)*182272i +
k^3*exp(26*k)*194880i - k^7*exp(22*k)*344064i - k^4*exp(26*k)*287616i - k^8*exp
(22*k)*147456i + k^5*exp(26*k)*494592i - k^9*exp(22*k)*655360i + k^2*exp(30*k)
*25200i - k^6*exp(26*k)*677888i - k^3*exp(30*k)*198912i + k^7*exp(26*k)*532480i +
k^4*exp(30*k)*389760i + k^8*exp(26*k)*81920i - k^5*exp(30*k)*537600i + k^9*exp(26*
k)*1310720i - k^2*exp(34*k)*22320i + k^6*exp(30*k)*1042432i + k^3*exp(34*k)*138048
i - k^7*exp(30*k)*655360i - k^4*exp(34*k)*328320i + k^8*exp(30*k)*81920i + k^5*exp
(34*k)*387072i - k^9*exp(30*k)*1310720i + k^2*exp(38*k)*13320i - k^6*exp(34*k)
*849920i - k^3*exp(38*k)*63552i + k^7*exp(34*k)*565248i + k^4*exp(38*k)*170688i -
k^8*exp(34*k)*147456i - k^5*exp(38*k)*178176i + k^9*exp(34*k)*655360i - k^2*exp
(42*k)*5160i + k^6*exp(38*k)*366592i + k^3*exp(42*k)*18208i - k^7*exp(38*k)*278528
i - k^4*exp(42*k)*50624i + k^8*exp(38*k)*81920i + k^5*exp(42*k)*47616i - k^9*exp
(38*k)*131072i + k^2*exp(46*k)*1176i - k^6*exp(42*k)*69632i - k^3*exp(46*k)*2816i
+ k^7*exp(42*k)*57344i + k^4*exp(46*k)*6720i - k^8*exp(42*k)*16384i - k^5*exp(46*k
)*5632i - k^2*exp(50*k)*120i + k^6*exp(46*k)*2048i + k^3*exp(50*k)*160i - k^4*exp
(50*k)*64i - k*exp(6*k)*48i + k*exp(10*k)*528i - k*exp(14*k)*2640i + k*exp(18*k)
*7920i - k*exp(22*k)*15840i + k*exp(26*k)*22176i - k*exp(30*k)*22176i + k*exp(34*k
)*15840i - k*exp(38*k)*7920i + k*exp(42*k)*2640i - k*exp(46*k)*528i + k*exp(50*k)
*48i - F*L*RM*RT*k^3*exp(6*k)*24i - F*L*RM*RT*k^4*exp(6*k)*192i + F*L*RM*RT*k^3*
exp(10*k)*240i + F*L*RM*RT*k^4*exp(10*k)*1536i - F*L*RM*RT*k^5*exp(10*k)*768i + F*
```

```
L*RM*RT*k^6*exp(10*k)*6144i - F*L*RM*RT*k^3*exp(14*k)*1056i - F*L*RM*RT*k^4*exp
(14*k)*4800i + F*L*RM*RT*k^5*exp(14*k)*7680i - F*L*RM*RT*k^6*exp(14*k)*36864i + F*
L*RM*RT*k^3*exp(18*k)*2640i + F*L*RM*RT*k^7*exp(14*k)*43008i + F*L*RM*RT*k^4*exp
(18*k)*5760i - F*L*RM*RT*k^8*exp(14*k)*49152i - F*L*RM*RT*k^5*exp(18*k)*34560i + F
*L*RM*RT*k^6*exp(18*k)*73728i - F*L*RM*RT*k^3*exp(22*k)*3960i - F*L*RM*RT*k^7*exp
(18*k)*307200i + F*L*RM*RT*k^4*exp(22*k)*5760i + F*L*RM*RT*k^8*exp(18*k)*196608i +
 F*L*RM*RT*k^5*exp(22*k)*92160i - F*L*RM*RT*k^9*exp(18*k)*393216i + F*L*RM*RT*k^3*
exp(26*k)*3168i + F*L*RM*RT*k^7*exp(22*k)*946176i - F*L*RM*RT*k^4*exp(26*k)*32256i
 - F*L*RM*RT*k^8*exp(22*k)*147456i - F*L*RM*RT*k^5*exp(26*k)*161280i + F*L*RM*RT*k
^9*exp(22*k)*1966080i - F*L*RM*RT*k^6*exp(26*k)*258048i - F*L*RM*RT*k^7*exp(26*k)
*1634304i + F*L*RM*RT*k^4*exp(30*k)*56448i - F*L*RM*RT*k^8*exp(26*k)*491520i + F*L
*RM*RT*k^5*exp(30*k)*193536i - F*L*RM*RT*k^9*exp(26*k)*3932160i + F*L*RM*RT*k^6*
exp(30*k)*516096i - F*L*RM*RT*k^3*exp(34*k)*3168i + F*L*RM*RT*k^7*exp(30*k)
*1720320i - F*L*RM*RT*k^4*exp(34*k)*57600i + F*L*RM*RT*k^8*exp(30*k)*1228800i - F*
L*RM*RT*k^5*exp(34*k)*161280i + F*L*RM*RT*k^9*exp(30*k)*3932160i - F*L*RM*RT*k^6*
exp(34*k)*516096i + F*L*RM*RT*k^3*exp(38*k)*3960i - F*L*RM*RT*k^7*exp(34*k)
*1118208i + F*L*RM*RT*k^4*exp(38*k)*37440i - F*L*RM*RT*k^8*exp(34*k)*1179648i + F*
L*RM*RT*k^5*exp(38*k)*92160i \textendash F*L*RM*RT*k^9*exp(34*k)*1966080i + F*L*RM
*RT*k^6*exp(38*k)*294912i - F*L*RM*RT*k^3*exp(42*k)*2640i + F*L*RM*RT*k^7*exp(38*k
)*430080i - F*L*RM*RT*k^4*exp(42*k)*15360i + F*L*RM*RT*k^8*exp(38*k)*540672i - F*L
*RM*RT*k^5*exp(42*k)*34560i + F*L*RM*RT*k^9*exp(38*k)*393216i - F*L*RM*RT*k^6*exp
(42*k)*92160i + F*L*RM*RT*k^3*exp(46*k)*1056i - F*L*RM*RT*k^7*exp(42*k)*86016i + F
*L*RM*RT*k^4*exp(46*k)*3648i - F*L*RM*RT*k^8*exp(42*k)*98304i + F*L*RM*RT*k^5*exp
(46*k)*7680i + F*L*RM*RT*k^6*exp(46*k)*12288i - F*L*RM*RT*k^3*exp(50*k)*240i + F*L
*RM*RT*k^7*exp(46*k)*6144i - F*L*RM*RT*k^4*exp(50*k)*384i - F*L*RM*RT*k^5*exp(50*k
)*768i + F*L*RM*RT*k^3*exp(54*k)*24i - F*L*RM*RT*V*k^3*exp(6*k)*12i - F*L*RM*RT*V*
k^4*exp(6*k)*96i + F*L*RM*RT*V*k^3*exp(10*k)*120i + F*L*RM*RT*V*k^4*exp(10*k)*864i
 + F*L*RM*RT*V*k^5*exp(10*k)*576i + F*L*RM*RT*V*k^6*exp(10*k)*4608i - F*L*RM*RT*V*
k^3*exp(14*k)*528i - F*L*RM*RT*V*k^4*exp(14*k)*3360i - F*L*RM*RT*V*k^5*exp(14*k)
*4608i - F*L*RM*RT*V*k^6*exp(14*k)*32256i + F*L*RM*RT*V*k^3*exp(18*k)*1320i - F*L*
RM*RT*V*k^7*exp(14*k)*9216i + F*L*RM*RT*V*k^4*exp(18*k)*7200i - F*L*RM*RT*V*k^8*
exp(14*k)*73728i + F*L*RM*RT*V*k^5*exp(18*k)*15552i + F*L*RM*RT*V*k^6*exp(18*k)
*92160i - F*L*RM*RT*V*k^3*exp(22*k)*1980i + F*L*RM*RT*V*k^7*exp(18*k)*55296i - F*L
*RM*RT*V*k^4*exp(22*k)*8640i + F*L*RM*RT*V*k^8*exp(18*k)*368640i - F*L*RM*RT*V*k
^5*exp(22*k)*27648i + F*L*RM*RT*V*k^9*exp(18*k)*49152i - F*L*RM*RT*V*k^6*exp(22*k)
*129024i + F*L*RM*RT*V*k^10*exp(18*k)*393216i + F*L*RM*RT*V*k^3*exp(26*k)*1584i -
F*L*RM*RT*V*k^7*exp(22*k)*129024i + F*L*RM*RT*V*k^4*exp(26*k)*4032i - F*L*RM*RT*V*
k^8*exp(22*k)*663552i + F*L*RM*RT*V*k^5*exp(26*k)*24192i - F*L*RM*RT*V*k^9*exp(22*
k)*196608i + F*L*RM*RT*V*k^6*exp(26*k)*64512i - F*L*RM*RT*V*k^10*exp(22*k)*1179648
i + F*L*RM*RT*V*k^7*exp(26*k)*129024i + F*L*RM*RT*V*k^4*exp(30*k)*4032i + F*L*RM*
RT*V*k^8*exp(26*k)*368640i + F*L*RM*RT*V*k^9*exp(26*k)*245760i + F*L*RM*RT*V*k^6*
exp(30*k)*64512i + F*L*RM*RT*V*k^10*exp(26*k)*786432i - F*L*RM*RT*V*k^3*exp(34*k)
*1584i - F*L*RM*RT*V*k^4*exp(34*k)*8640i + F*L*RM*RT*V*k^8*exp(30*k)*368640i - F*L
*RM*RT*V*k^5*exp(34*k)*24192i - F*L*RM*RT*V*k^6*exp(34*k)*129024i + F*L*RM*RT*V*k
^10*exp(30*k)*786432i + F*L*RM*RT*V*k^3*exp(38*k)*1980i - F*L*RM*RT*V*k^7*exp(34*k
)*129024i + F*L*RM*RT*V*k^4*exp(38*k)*7200i - F*L*RM*RT*V*k^8*exp(34*k)*663552i +
F*L*RM*RT*V*k^5*exp(38*k)*27648i - F*L*RM*RT*V*k^9*exp(34*k)*245760i + F*L*RM*RT*V
*k^6*exp(38*k)*92160i - F*L*RM*RT*V*k^10*exp(34*k)*1179648i - F*L*RM*RT*V*k^3*exp
(42*k)*1320i + F*L*RM*RT*V*k^7*exp(38*k)*129024i - F*L*RM*RT*V*k^4*exp(42*k)*3360i
 + F*L*RM*RT*V*k^8*exp(38*k)*368640i - F*L*RM*RT*V*k^5*exp(42*k)*15552i + F*L*RM*
RT*V*k^9*exp(38*k)*196608i - F*L*RM*RT*V*k^6*exp(42*k)*32256i + F*L*RM*RT*V*k^10*
exp(38*k)*393216i + F*L*RM*RT*V*k^3*exp(46*k)*528i - F*L*RM*RT*V*k^7*exp(42*k)
*55296i + F*L*RM*RT*V*k^4*exp(46*k)*864i - F*L*RM*RT*V*k^8*exp(42*k)*73728i + F*L*
RM*RT*V*k^5*exp(46*k)*4608i - F*L*RM*RT*V*k^9*exp(42*k)*49152i + F*L*RM*RT*V*k^6*
```

```
exp(46*k)*4608i - F*L*RM*RT*V*k^3*exp(50*k)*120i + F*L*RM*RT*V*k^7*exp(46*k)*9216i
 - F*L*RM*RT*V*k^4*exp(50*k)*96i - F*L*RM*RT*V*k^5*exp(50*k)*576i + F*L*RM*RT*V*k
^3*exp(54*k)*12i))/(12*k^3*(exp(-2*k) - exp(2*k))*(exp(4*k) - 1)^2*(2*exp(4*k) -
exp(8*k) + 16*k^2*exp(4*k) - 1)*(exp(4*k) - 4*exp(8*k) + 6*exp(12*k) - 4*exp(16*k)
 + exp(20*k) - 16*k^2*exp(8*k) + 32*k^2*exp(12*k) - 16*k^2*exp(16*k))*(4*exp(4*k)
- 6*exp(8*k) + 4*exp(12*k) - exp(16*k) + 16*k^2*exp(4*k) - 32*k^2*exp(8*k) + 16*k
^2*exp(12*k) - 1))
```
